# Neuroinflammation-Associated Aspecific Manipulation of Mouse Predator Fear by *Toxoplasma gondii*

**DOI:** 10.1016/j.celrep.2019.12.019

**Published:** 2020-01-14

**Authors:** Madlaina Boillat, Pierre-Mehdi Hammoudi, Sunil Kumar Dogga, Stéphane Pagès, Maged Goubran, Ivan Rodriguez, Dominique Soldati-Favre

**Affiliations:** 1 Department of Genetics and Evolution, Faculty of Sciences, University of Geneva, 1211 Geneva, Switzerland; 2 Department of Microbiology and Molecular Medicine, Faculty of Medicine-University of Geneva CMU, 1 rue Michel-Servet 1211 Geneva 4, Switzerland; 3 Wyss Center for Bio- and Neuroengineering, Geneva, Switzerland; 4 Department of Basic Neurosciences, University of Geneva, Geneva, Switzerland; 5 Hurvitz Brain Sciences Program, Sunnybrook Research Institute, University of Toronto, Toronto, ON, Canada

**Keywords:** *Apicomplexa*, *Toxoplasma gondii*, parasites, chronic infection, host-pathogen interaction, effector molecules, innate behavior, predator avoidance, light-sheet microscopy, cat

## Abstract

In rodents, the decrease of felid aversion induced by *Toxoplasma gondii*, a phenomenon termed fatal attraction, is interpreted as an adaptive manipulation by the neurotropic protozoan parasite. With the aim of understanding how the parasite induces such specific behavioral modifications, we performed a multiparametric analysis of *T. gondii*-induced changes on host behavior, physiology, and brain transcriptome as well as parasite cyst load and distribution. Using a set of complementary behavioral tests, we provide strong evidence that *T. gondii* lowers general anxiety in infected mice, increases explorative behaviors, and surprisingly alters predator aversion without selectivity toward felids. Furthermore, we show a positive correlation between the severity of the behavioral alterations and the cyst load, which indirectly reflects the level of inflammation during brain colonization. Taken together, these findings refute the myth of a selective loss of cat fear in *T. gondii*-infected mice and point toward widespread immune-related alterations of behaviors.

## Highlights

• *Toxoplasma gondii* has a widespread impact on rodent behavior
• Infection-induced loss of fear is not specific to feline predators
• Behavioral alterations directly correlate with cyst burden and neuroinflammation
• Cysts are predominantly localized in cortical areas of the brain

## Graphical Abstract

**Figure:**
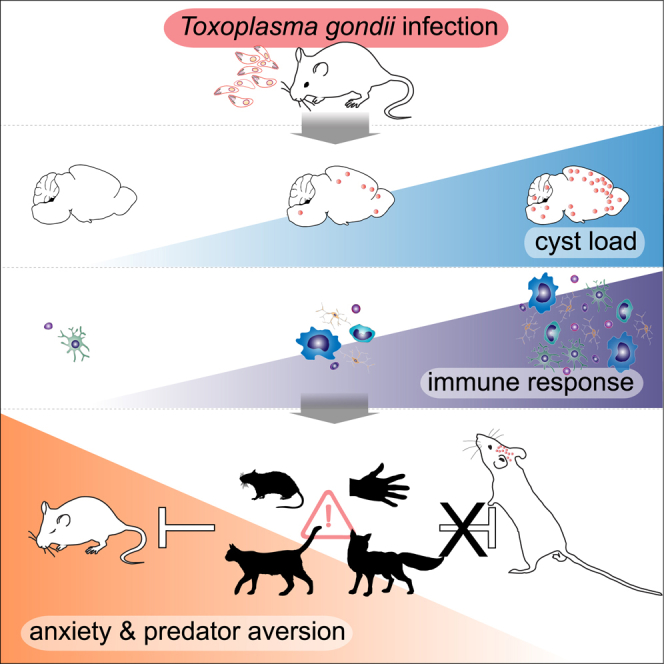

## Teaser

Contradicting the prevailing model of a selective loss of cat fear in *Toxoplasma gondii*-infected rodents, Boillat et al. show in a multiparametric analysis of host behavior, physiology, and brain transcriptome that the loss of predator fear is not specific to felids and that the severity of behavioral alterations correlates with neuroinflammation.

## Introduction

Innate behaviors are determinant for the survival of animals. Among these critical predetermined reactions, prey have a vital necessity to display appropriate responses, like freezing or fleeing, the very first time they encounter a predator. Thus, instinctive predator avoidance represents a survival strategy whose alteration may greatly affect the survival chances of prey.

*Toxoplasma gondii* is a pathogen whose life cycle is at the crossroads of predator-prey interplay. Member of the Apicomplexa phylum, *T. gondii* displays an obligatory intracellular lifestyle and replicates sexually within the intestinal tract of felids, its definitive hosts. Felids are infected by tissue cysts when they scavenge an infected prey. The parasite undergoes gametogenesis in the felid intestine, and the infectious oocysts resulting from sexual reproduction are shed off by the feces into the environment. These oocysts are taken up by warm-blooded animals, including mammals, marsupials, and birds, constituting a very large pool of intermediate hosts (Lindsay and Dubey, 2014). Once ingested, the sporozoites are freed from within the oocyst by proteolytic enzymes in the stomach and small intestine and then converted to the fast-replicative tachyzoites that disseminate in all organs. This causes an acute infection of variable severity depending on the host susceptibility and immune status. In immunocompetent hosts, tachyzoites rapidly differentiate into slow-growing bradyzoites enclosed within tissue cysts that persist in long-lived cells, predominantly neurons and skeletal muscle cells (Dzierszinski et al., 2004, Radke et al., 2003). Although the acute phase of *T. gondii* infection is generally asymptomatic, behavioral changes have been associated with chronic infection in various hosts (Berdoy et al., 2000, Webster, 2007, Webster et al., 2013). With a worldwide prevalence of about 30% in the human population, latent toxoplasmosis is a risk factor for several mental illnesses, including schizophrenia, Parkinson’s disease, and bipolar disorders (Fabiani et al., 2015). In rodents, *T. gondii* is known to alter the innate aversion to felid odors (Berdoy et al., 2000, Hammoudi and Soldati-Favre, 2017, Vyas, 2015, Vyas et al., 2007). This latter aspect of *T. gondii* parasitism is referred to as the “fatal attraction phenomenon,” which refers to the ability of the parasite to manipulate its intermediate host that becomes specifically attracted to felids, hence facilitating parasite spreading. This postulate received great attention and is supported by studies showing a decreased aversion to felid odors by *T. gondii*-infected rodents (Berdoy et al., 2000, Kannan et al., 2010, Lamberton et al., 2008, Vyas et al., 2007). The decreased aversion to felids is seemingly not resulting from an impairment of olfactory faculties (Berdoy et al., 1995, Torres et al., 2018) but appears to be a consequence of complex neuronal and physiological mechanisms altering the perception of the host when facing a predation risk. Multiple neuronal changes that occur during infection, such as the alteration of neurotransmitter levels or metabolism (Alsaady et al., 2019, Ihara et al., 2016, Skallová et al., 2006, Webster et al., 2006, Xiao et al., 2016) and hormone level changes (Hari Dass and Vyas, 2014), were proposed to be involved in the modulation of behavior. Nevertheless, how these parameters alter the behavioral response to felids and, importantly, how specificity to this mammalian family is achieved remain elusive.

At the periphery, the neural circuitry for predator aversion in rodents involves sensory neurons expressing specific olfactory receptors that recognize determined predatory kairomones (Dewan et al., 2013, Ferrero et al., 2011, Papes et al., 2010, Samuelsen and Meredith, 2009). The signals then converge in the ventromedial hypothalamus (VMH) or the amygdala, which mediate behavioral responses (Papes et al., 2010, Pérez-Gómez et al., 2015, Takahashi, 2014). Various conflicting results regarding a potential specific tropism of *T. gondii* cyst location toward these two regions of the brain have been published (Afonso et al., 2012, Berenreiterová et al., 2011, Dellacasa-Lindberg et al., 2007, Dubey et al., 2016, Evans et al., 2014, Gonzalez et al., 2007, Haroon et al., 2012, Hermes et al., 2008, Tanaka et al., 2013, Vyas et al., 2007). A majority of the studies agree on a widespread but non-homogeneous distribution of cysts in different brain areas, but no consensus on which regions are preferentially enriched in cysts has been reached. Considering these existing data, the specific effect on felid cue processing is hardly explained. Similarly, many contradictory or inconsistent results have been reported relative to effects of *T. gondii* infection on rodent behavior (Worth et al., 2013, Worth et al., 2014), making conclusions on the underlying mechanisms challenging.

The aim of this work was to delineate the range and origin of behavioral alterations induced by *T. gondii* infection in rodents by performing a multiparametric study. Our approach involved a large battery of behavioral assays, comprising both standard and newly established tests. We found that the loss of predator fear in *T. gondii*-infected mice is not specific to felid predators and confirmed that a broad range of non-predator-related behaviors are also strongly affected. The impact of infection on behavior was reduced in mice infected with mutant parasite strains with low virulence and with strains defective in the export of effector proteins into host cells. Cyst load, markers of inflammation, and transcriptional changes occurring in the host brain indicated that the severity of the behavioral alterations is associated with neuroinflammation. Additionally, we established a tool to accurately map the brain-wide location and number of cysts at an unprecedented resolution in 3D, which highlights widespread localization affecting predominantly cortical areas. Together, our observations contradict the prevailing model of a selective loss of cat fear in *T. gondii*-infected rodents.

## Results

### *Toxoplasma gondii* Chronic Infection Triggers a Decrease in Anxiety and Enhances Exploration

**Figure 1:**
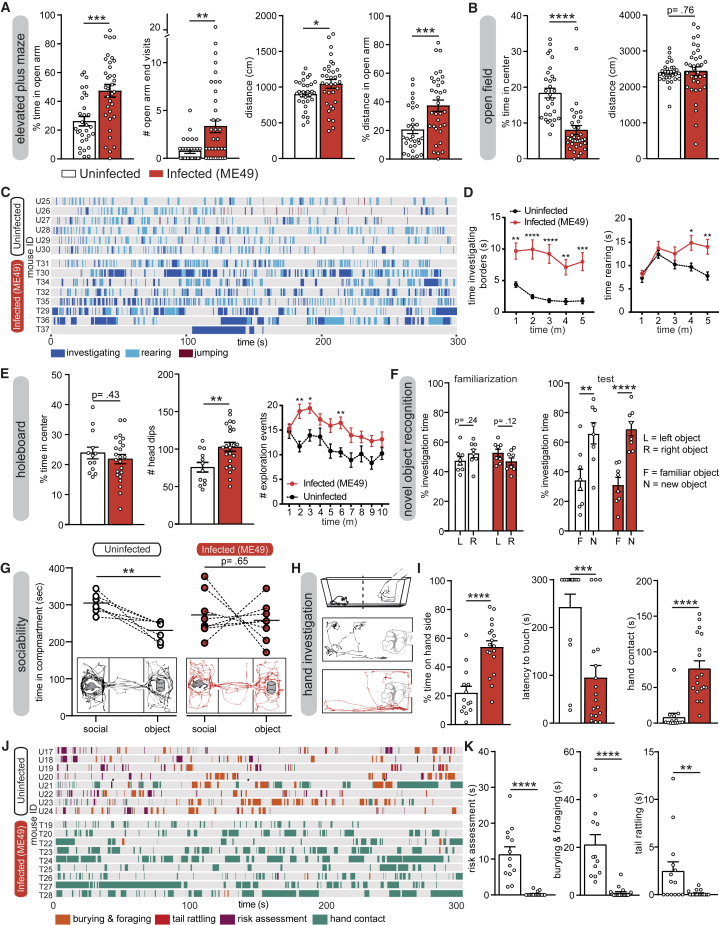
*T. gondii* Infection Leads to Decreased Anxiety and Increased Exploration Behaviors (A and B) Quantification of behaviors of uninfected and *T. gondii* ME49-infected mice in the EPM (A) and in the OF (B). (C) Ethogram showing explorative behaviors (investigation of borders, rearing) and escape behavior (jumping) of a representative sample of analyzed mice in the OF. (D) Each line represents one individual. Explorative behaviors from all mice were quantified and plotted as a function of time. (E) Quantification of explorative behaviors in the holeboard test. Each dot represents one individual except for the time course graphs where mean ± SEM are represented. Exploration events comprise head dips and rearing. (F) Evaluation of short-term memory in an object recognition test. Investigation preferences are quantified during a familiarization session (two identical objects) and a test session (one familiar and one new object). (G) Time spent in each side compartment of the three-chamber arena containing a social stimulus or an object. Each individual is represented by two connected points; horizontal lines represent means. Illustrations on the bottom show example traces from an uninfected and a *T. gondii-*infected mouse. (H) Schematic of hand investigation test and example traces from an uninfected (black) and a *T. gondii*-infected mouse (red). (I) Quantification of behaviors in the hand investigation test. (J) Ethogram showing defensive and anxiety-related behaviors (burying of the hand and foraging, tail rattling, and risk assessment) and explorative behaviors (hand contact). Each line represents one individual. (K) Quantification of defensive behaviors during the hand investigation test. Uninfected, n = 7–30; infected (ME49), n = 8–37. Bars indicate mean ± SEM, and each dot represents an individual. ^∗^p < 0.05, ^∗∗^p < 0.01, ^∗∗∗^p < 0.001, ^∗∗∗∗^p < 0.0001. For details of the statistical analyses, see Table S1. For detailed results of behavioral assays, see Table S2.

To get a clear overview of how *T. gondii* affects host behavior in general, infected mice went through a series of behavioral tests (Figure S1A; Table S2). For all experiments described in this study, male B6CBAF1/J mice were infected by intraperitoneal (i.p.) injection of the indicated parasite strain. They were evaluated during the chronic infection phase, from 5 weeks to 10 weeks post-infection (p.i.). To assess the overall anxiety level of infected mice, animals were tested in an elevated plus maze (EPM). We observed a significant increase in the time spent in the open arms by infected mice compared to the uninfected group, suggesting a reduced anxiety in infected mice (Figures 1A and S1B). The mean number of visits at the end of the open arms also differed. The total distance traveled was slightly higher in infected animals and, in particular, the percent distance covered in the open arm. The basal anxiety level was also investigated in an open-field (OF) arena (Figures 1B and S1C). We observed that infected mice spent less time than uninfected mice in the central square of the OF, a result initially surprising because it suggests an increase of anxiety level in infected mice, in apparent contradiction with the EPM results. However, a more detailed analysis of behaviors during the OF test highlighted a marked rise of exploratory behaviors of infected mice on the borders of the arena, in particular rearing and olfactory investigation (Figures 1C and 1D). Interestingly, uninfected mice decreased their exploratory behaviors over time, whereas exploration remained high in infected mice. No difference in the distance covered by animals of the uninfected and infected groups was noticed (Figure 1B). Performed together, the OF and the EPM are showing consistent outputs that clarify the conflicting data regarding these two tests previously reported (Worth et al., 2014). To further characterize the effect of *T. gondii* infection on explorative behaviors, we evaluated the interaction of mice with different non-threatening stimuli (Figures S1D–S1F). Mice were allowed to freely investigate previously unknown stimuli in an open arena: a metallic cube with holes, an apple, and an unfamiliar non-aggressive male mouse (Figure S1D). No difference in total interaction time was observed, and uninfected mice showed an expected and clear preference for investigating conspecifics rather than inert objects (Figures S1E and S1F). In contrast, *T. gondii*-infected mice showed a high interest in all three stimuli. Next, we specifically evaluated the exploration of a novel environment by performing a holeboard test (Boissier et al., 1964). Infected mice showed significantly more exploratory behavior than uninfected ones, in particular through head-dipping (Figures 1E and S1G). This increased exploration by infected mice (also observed in the OF assay) may reflect a deficit in short-term memory, preventing habituation to the environment. To evaluate this possibility, we performed a novel object recognition test (Figure 1F). During the familiarization session, where mice were exposed to two identical objects, uninfected and infected mice investigated both objects equally (Figure 1F). During the test session, where mice were exposed to a familiar and a novel object, both uninfected and infected mice spent significantly more time investigating the novel object, hence excluding the short-term memory impairment hypothesis.

Our initial interaction test (Figures S1D–S1F) suggested that infected mice might display altered sociability. To further explore this observation, we performed a sociability test where the propensity of the animal to investigate a neutral object or a social stimulus was measured (Figure 1G). Uninfected mice showed a clear preference for the social stimulus, whereas infected mice showed no preference for any of the stimuli. We next asked whether *T. gondii* infection could affect the way animals interact with a potentially threatening stimulus. With this aim, we designed a novel assay—the hand investigation assay—that measured the propensity of a naive animal to interact with the hand of an experimenter (Figure 1H). As opposed to sham-infected mice, *T. gondii*-infected animals did not avoid the cage side on which the hand was present; on the contrary, they quickly touched the hand and thoroughly interacted with it (Figures 1I and 1J). During the assay, uninfected mice displayed significantly more defensive and anxiety-related behaviors than infected mice, such as risk assessment episodes, foraging or burying the hand, and tail rattling (Figures 1J and 1K). Finally, to evaluate whether the observed changes in behavior could result from differences in basal levels of stress, we measured the plasmatic concentration of stress hormones. No clear differences were observed, besides a tendency for slightly lower adrenocorticotropic hormone (ACTH) concentrations in infected mice (Figure S1G).

Collectively, these findings demonstrate that animals chronically infected by *T. gondii* display reduced anxiety levels and risk aversion, as well as increased curiosity and exploration, when put in challenging situations. Furthermore, infected mice do not discriminate between live and inert stimuli. Taken together, these observations suggest an altered ability to adequately process signals that may represent potential threats.

### *Toxoplasma gondii* Infection Does Not Selectively Alter Fear of Felid Predators

**Figure 2:**
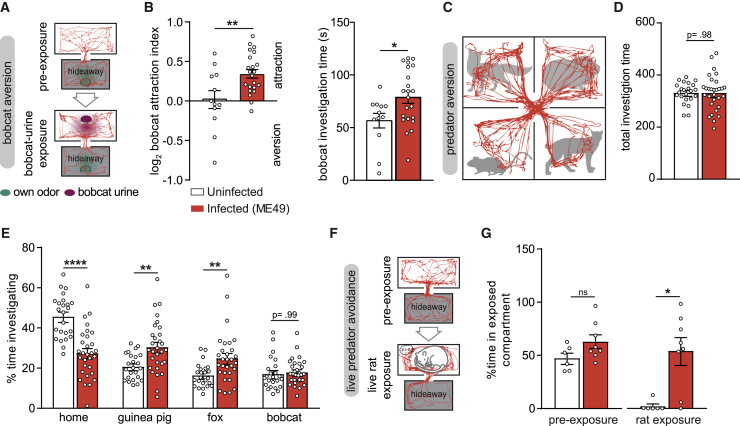
Non-specific Loss of Predator Aversion in *T. gondii*-Infected Mice (A) Schematic of bobcat aversion test with example traces from a mouse infected with *T. gondii*. (B) Quantification of behaviors during bobcat aversion test. Bobcat aversion index is the (time spent in the open compartment with bobcat odor)/(time spent in open compartment without bobcat odor). Positive values signify increased presence in open compartment when the odor is present. (C) Schematic of predator avoidance test in a 4-chamber arena containing two predator odors (fox and bobcat), a non-predator odor (guinea pig), and the odor of the mouse (home). Representative trace from a *T. gondii* ME49-infected mouse. (D and E) Quantification of total investigation times (D) and investigation times of each odor source (E). (F) Schematic of live predator avoidance test in a two-chamber arena with a hideaway and an exposed compartment containing an anesthetized rat. Representative traces from a *T. gondii* ME49-infected mouse. (G) Quantification of percentage of time spent in the exposed compartment before and after introduction of the rat. Bars indicate mean ± SEM, and each dot represents an individual. ^∗^p < 0.05, ^∗∗^p < 0.01, ^∗∗∗^p < 0.001, ^∗∗∗∗^p < 0.0001. For details of the statistical analyses, see Table S1.

One of the most publicized effects of *T. gondii* on rodent behavior is the unusual attraction for felid odors that chronically infected animals exhibit (Berdoy et al., 2000, Vyas et al., 2007). Nevertheless, several gaps and inconsistencies among reported studies (Worth et al., 2014) encouraged us to reappraise this dogma. We first evaluated whether infected mice display an attraction toward felid predator urine (bobcat) in a simple two-chamber arena comprising a hideaway and an exposed compartment (Figure 2A). Uninfected mice responded variably to bobcat odor, whereas a clear attraction was observed in *T. gondii*-infected mice (Figure 2B). Next, to address the specificity of this behavioral subversion, we tested mice in an experimental set-up that included a second predator odor (fox) as well as a non-predator odor (guinea pig) (Figure 2C). Control mice spent most of the time in the compartment containing their own odor and investigated almost equally the three other compartments, showing a weak but non-significant preference for the guinea pig odor (Figures 2E and S2A). On the other hand, *T. gondii*-infected mice were more willing to leave the compartment containing their own odor, and spent more time exploring those containing the guinea pig odor and fox odor. No particular attraction to bobcat odor was observed when infected mice had the choice between multiple complex odors. Of note, no difference was observed between mice from both groups in the total time investigating the odors (Figure 2D), indicating that the increased time spent investigating predator odors does not simply result from increased exploration.

As mice quickly habituate to predator odors in laboratory experimental set-ups, displaying limited fear, we expanded our observations to a more natural setting, which involved a live predator. For this purpose, mice were allowed to interact with an anesthetized rat in a two-chamber arena (Figure 2F). During the pre-exposure session, control and infected mice spent a similar amount of time in the exposed compartment (Figure 2G). Markedly, when a rat was introduced, control mice remained confined in the hideaway, whereas infected mice were more inclined to spend time in the compartment with the rat (Figures 2F, 2G, and S2B).

Together, these results show that *T. gondii*-infected mice exhibit a general loss of predator avoidance behavior, which is not specific to felid predators, the definitive host of the parasite. These results contrast with the prevailing idea that the parasite manipulation of host behavior specifically targets neural circuits responding to felid predators.

### Severity of Behavioral Changes Correlates with the Cyst Burden

**Figure 3:**
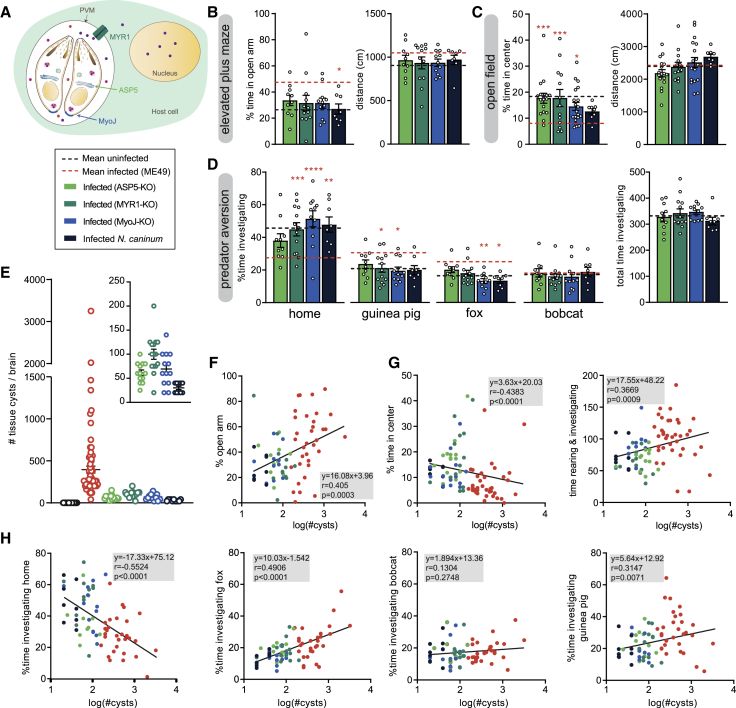
Severity of Behavioral Changes Induced by *T. gondii* Infection Correlates with Cyst Load (A) Schematic of a *T. gondii*-infected cell illustrating different components involved in protein export from parasite to host cell and in parasite virulence. Colored dots represent dense granule proteins. PVM, parasitophorous vacuole membrane. (B) Quantification of behaviors of mice infected with *T. gondii* parasites deficient for protein export (ASP5-KO and MYR1-KO), with lower cyst number (MyoJ-KO) or with a different apicomplexan parasite (*N. caninum*) in the EPM. Behavioral phenotype is compared to means of uninfected (black) and ME49-infected mice (red), which are represented by dotted lines. (C) Quantification of behaviors in the OF. (D) Quantification of investigation times in the predator avoidance assay. (E) Cyst counts from all tested individuals. Insert on top right corner shows cyst counts only from low-cyst variants. (F) Correlation of anxiety (% time spent in open arm in EPM) and cyst load. (G) Correlation of explorative behaviors (rearing + investigation) in the OF and cyst load. (H) Correlation between investigation times of different odors in the predator aversion test and cyst load. (A–D) Bars indicate mean ± SEM, and each dot represents an individual. ^∗^p < 0.05, ^∗∗^p < 0.01, ^∗∗∗^p < 0.001, ^∗∗∗∗^p < 0.0001; pink asterisks represent significant differences compared to ME49-infected mice. Each dot represents one individual; parasite genotype is represented by the color. For details of the statistical analyses, see Table S1.

We next investigated whether the origin of the behavioral modulation of infected hosts could result from parasite-secreted effector proteins. We infected mice with two different mutant strains of *T. gondii*, aspartyl protease 5 knockout (ASP5-KO) and Myc regulation 1 knockout (MYR1-KO) (Coffey et al., 2015, Curt-Varesano et al., 2016, Franco et al., 2016, Hammoudi et al., 2015), where protein export across the parasitophorous vacuole membrane (PVM) is compromised (Figures 3A and S3A–S3C; Table S4), and analyzed their behavior. In the EPM and OF, as well as in the predator avoidance test, mice infected with the mutant strains either displayed behaviors similar to uninfected mice or intermediate between uninfected and ME49-infected mice (Figures 3B–3D and S3D–S3E). Nevertheless, in the absence of protein export, parasite spreading and virulence is also severly affected during acute infection (Franco et al., 2016, Hammoudi et al., 2015), leading to a drastic drop in tissue-cyst number in the brain of ASP5-KO- and MYR1-KO-infected mice (Figure 3E). To assess whether the marked difference in severity of behavioral alterations between the mutants and the wild-type ME49 infection resulted from reduced cyst load or from the defect in protein export, we also analyzed the behavior of mice infected with myosin J knockout (MyoJ-KO) parasites that lead to a reduced number of cysts but has no impact on protein export (Frénal et al., 2017). In addition, we analyzed mice infected with the closely related apicomplexan parasite *Neospora caninum* whose definitive host are canids (Arranz-Solís et al., 2015). As for the ASP5-KO- and MYR1-KO-infected mice, MyoJ-KO- and *N. caninum*-infected mice exhibited similar anxiety and exploration profiles as uninfected mice and displayed clear predator avoidance (Figures 3B–3D and S3D–S3E). These observations suggest that the severity of the infection and the cyst load are determinants for the severity of the behavioral alterations of infected mice. Given the large inter-individual behavioral variations observed among infected mice, we tested a potential correlation between cyst load and behavioral phenotype, independent of parasite genotype. We found a positive correlation between cyst load and time spent in the open arm of the EPM (Figure 3F) as well as the amount of explorative behaviors in the OF (Figure 3G). In the predator avoidance test, we observed a striking negative correlation between the time spent in the home compartment and the cyst load and a positive correlation for the guinea pig and fox compartments but not for the bobcat compartment (Figure 3H).

Taken together, these results establish that cyst load is associated with the severity of *T. gondii*-induced behavioral alterations in mice. They also indicate that exported effector proteins have a strong impact on parasite virulence and persistence, which indirectly affect behavior.

### *Toxoplasma gondii* Sustainably Alters the Host Brain Transcriptome and, in Particular, Immune-Related Genes

**Figure 4:**
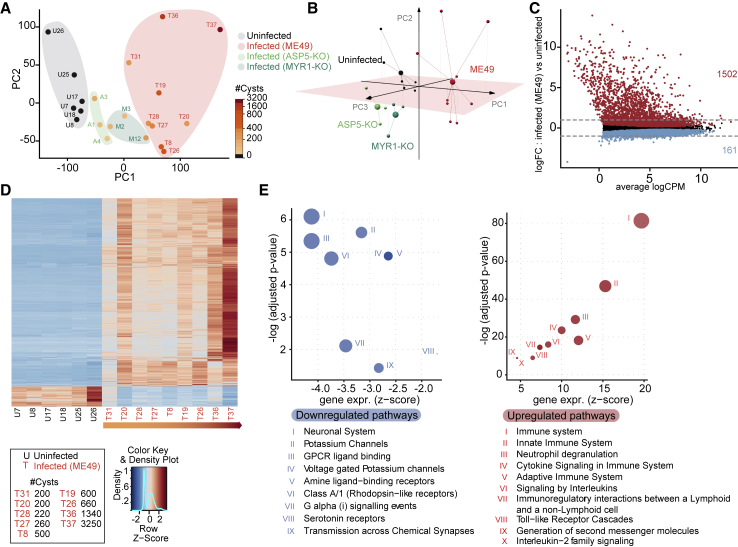
Host Brain Transcriptome Shows Sustained Immune Response in the CNS (A) 2D PCA representing the differences between individual mice analyzed by RNA-seq. The cyst load in infected mice is indicated with a color scale. Background colors show to which infection condition individuals pertain. (B) 3D PCA highlighting the differences between uninfected and infected individuals. (C) Differential expression (DE) analysis showing expression of all detectable genes. Red dots indicate upregulated genes; blue dots indicate downregulated genes (FDR < 0.05). Dashed lines indicate the threshold of a 2× fold change (FC). (D) Heatmap displaying the expression of all up- and downregulated genes (FC 2) across the 6 uninfected and the 9 *T. gondii* ME49-infected mice. Individuals are ordered according to cyst load. (E) Pathway enrichment analysis for up- and downregulated genes in mice infected with *T. gondii* ME49.

To assess the impact of *T. gondii* infection as well as the importance of the exported effector proteins on the host transcriptome during chronic infection, we performed high-throughput RNA sequencing on mouse brains harvested 10–12 weeks p.i. RNA was prepared from the whole brain of uninfected, ME49-, ASP5-KO-, and MYR1-KO-infected mice previously tested for behavior and assessed for cyst load. To visualize the similarities between transcriptomes and to identify putative vectors of differentiation, we computed principal-component analysis (PCA) by using the expression data of all genes detected in uninfected and infected samples. The 2D projections of the samples using the two first principal components, which explain ∼60% of the variance, highlighted clear distinctions between the four groups (Figure 4A), with cyst load seemingly impacting on the PC1 separation. Using the three first principal components, explaining ∼70% of the variance, we found that 3D projections emphasized the differences between the four groups and, in particular, distinguished mice infected with ASP5-KO and MYR1-KO parasites from the ME49-infected and uninfected mice clusters (Figure 4B). We then performed a differential expression (DE) analysis with a false discovery rate (FDR) set at 5%, in addition to a 2-fold change threshold, to detect upregulated or downregulated genes between uninfected and ME49-infected mice with varying cyst load. Using the above cut-off criteria, we found 1,663 host genes to be significantly differentially expressed, representing ∼11% of the ∼14,500 detected genes (Figure 4C), with a majority being upregulated (Figure 4D). Pathway enrichment analysis revealed that a substantial number of genes related to immunity were upregulated in the brains of infected mice (Figure 4E). Downregulated genes were mainly involved in pathways related to neuronal signaling (Figure 4E).

### Gene Expression Levels Are Associated with Cyst Load and with Behavioral Alterations

**Figure 5:**
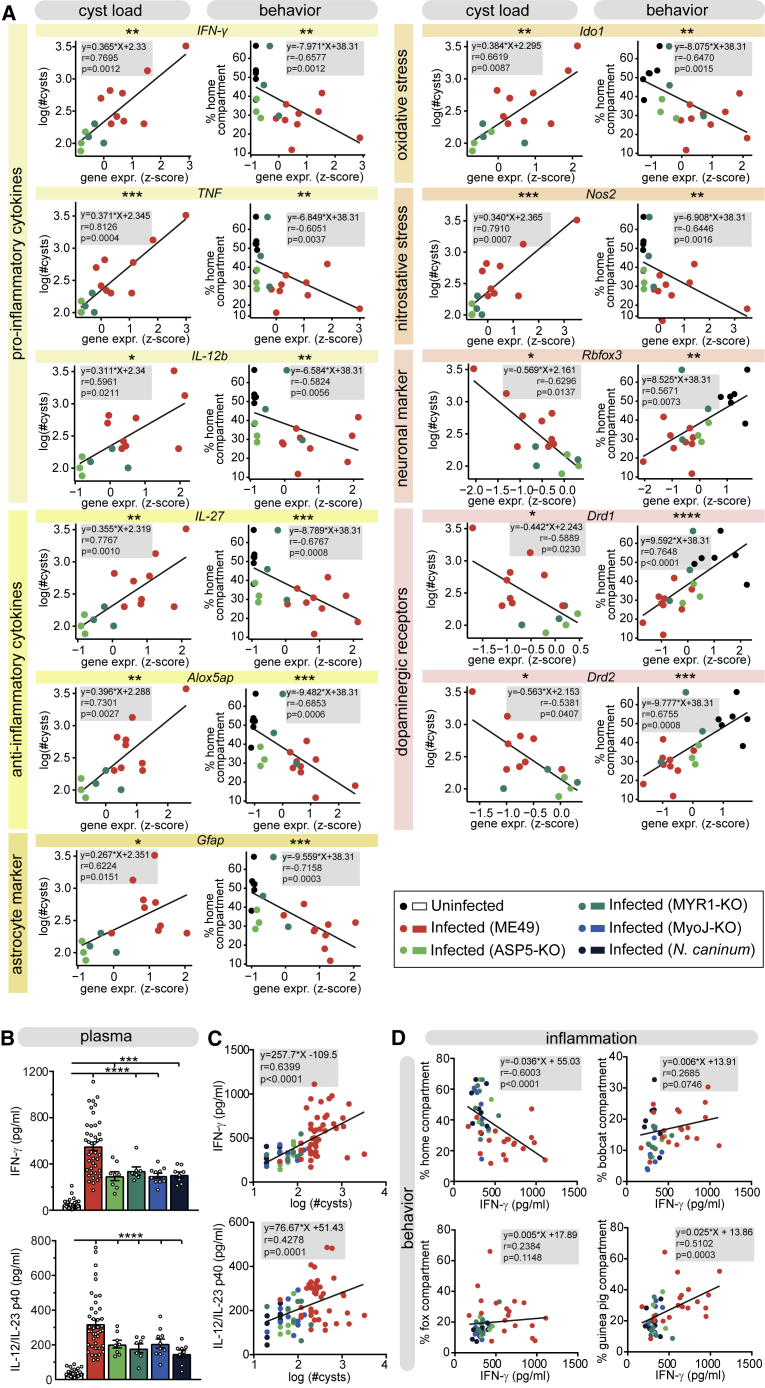
Association of Gene Expression Levels with Infection and Behavior (A) Correlations between cyst load (left panels), a behavioral score (the time spent in the home compartment during the 4-chamber predator aversion test; right panels), and relative gene expression levels representative of the up- and downregulated pathways (see also Figure S5A). Each dot represents one individual. (B) Concentration of IFN-γ and IL-12/IL-23 p40 in the plasma at 7–10 weeks p.i. of mice infected by the indicated parasite strain. Bars indicate mean ± SEM, and each dot represents an individual. ^∗∗∗^p < 0.001, ^∗∗∗∗^p < 0.0001. (C) Correlations between the concentration of IFN-γ and IL-12/IL-23 p40 in the plasma and cyst load. (D) Correlations between investigation times of different odors in the predator aversion test and the concentration of IFN-γ and IL-12/IL-23 p40 in the plasma. Each dot represents one individual; parasite strain and variant is represented by the color. For correlations, ^∗^p < 0.5, ^∗∗^p < 0.01, ^∗∗∗^p < 0.001, ^∗∗∗∗^p < 0.0001, Spearman’s correlation. For details of the statistical analyses, see Table S1.

We then looked at the expression levels of individual genes representative of the identified altered pathways, and genes that are either known to be involved in the inflammatory response or known to alter neuronal function upon *T. gondii* infection (Figures 5A and S5A). First, we evaluated the association between gene expression levels and cyst load (Figure 5A, left panels). Second, we tested potential correlations between gene expression levels and the severity of a behavioral alteration, notably the time spent in the home compartment during the 4-chamber predator aversion test (Figure 5A, right panels). Resistance to *T. gondii*, defined by the control of growth and replication as well as the promotion of intracellular mechanisms to kill the parasite, is mediated through pro-inflammatory cytokines (Däubener et al., 1996, Gazzinelli et al., 1993, Suzuki et al., 1988). We observed that both cyst load and behavior were correlated with the expression levels of interferon-gamma (*IFN-γ*), interleukin-12b (*IL-12b*), and tumor necrosis factor (*TNF*) (Figure 5A; Figure S5A). Genes with antagonist functions like *IL-27* and *Alox5ap*, which help to control the immune response (Aliberti et al., 2002, Villarino et al., 2003), were also upregulated in highly parasitized brains and correlated with the behavioral alteration. Overexpression of *Gfap*, a marker for astrocytes, also reflected the neuroinflammation occurring in highly infected brains and correlated with behavior (Pekny and Pekna, 2004). The association between *T. gondii* infection and neuropsychiatric disorders has previously been suggested to result, at least in part, from the cytokine-mediated activation of indoleamine-2,3-dioxygenase (IDO), which induces tryptophan degradation, leading to increased levels of neuroactive metabolites that can disturb glutamatergic and dopaminergic neurotransmission (Donley et al., 2016, Lugo-Huitrón et al., 2013, Schwarcz et al., 2001). Relevantly, *Ido1* transcription was upregulated in infected mice, as well as another downstream effector of IFN*-γ,* the nitric oxide synthase (*Nos2*). Despite their protective role against the parasite, high production of nitric oxide can also lead to neuronal toxicity through nitrosative and oxidative stress (Beckman and Koppenol, 1996, Erusalimsky and Moncada, 2007). Expression levels of *Ido1* and *Nos2* were also positively correlated with the behavioral alteration. *Rbfox3*, a general neuronal marker, which was also associated with changes in behavior, displayed lower expression in highly infected brains, probably reflecting both neuronal loss and massive infiltration of non-neuronal cells in the CNS. Among the downregulated genes, we noticed an expected strong decrease in the expression of dopamine receptor *Drd1* and *Drd2* genes in mice with high cyst burden. The level of expression of both dopaminergic receptors strongly correlated with behavioral alterations.

Together, these observations indicate that persistent *T. gondii* cysts in the CNS maintain a sustained immune response and perturb prominent neuronal functions associated with behavioral disorders in chronically infected animals, strengthening and expanding previous publications (Alsaady et al., 2019, Garfoot et al., 2019, Pittman et al., 2014, Tanaka et al., 2013, Wang et al., 2018, Xiao et al., 2012). Strikingly, we observed that gene expression levels corresponding to specific pathways were strongly associated with changes in behavior.

### The Concentration of Inflammation Markers Reflects the Degree of Behavioral Alterations

Analyses of the host transcriptomic profiles during chronic infection showed a positive association between the expression of many immune- and neuronal-related genes and cyst load. We sought to evaluate a potential direct correlation between the response of immune players and behavioral alterations. We first measured the level of pro-inflammatory cytokines secreted upon *T. gondii* infection by detecting them in the plasma at different time points during both acute and chronic infection. Concentrations of IFN-γ and IL-12/IL-23 p40 rose quickly after infection and then remained high at all subsequent time points, which reflected the level of inflammation endured by animals infected with *T. gondii* (Figure S5B). We assessed the concentration of IFN-γ and IL-12/IL-23 p40 in the plasma at 7–10 weeks p.i. and observed intermediate levels in ASP5-KO-, MYR1-KO-, MyoJ-KO-, and *N. caninum*-infected mice compared to uninfected and ME49-infected mice (Figure 5B). However, we found a fairly wide inter-individual distribution of cytokine levels among ME49-infected mice, suggesting a possible cyst-load-dependent effect. Indeed, we observed a clear positive correlation between IFN-γ and IL-12/IL-23 p40 levels and cyst load, independent of parasite genotype (Figure 5C). Furthermore, we questioned whether pro-inflammatory cytokines levels correlated with the severity of the behavioral changes. In the predator avoidance test, we noticed a marked negative correlation between the time spent in the home compartment and IFN-γ levels, pointing to a direct link between the degree of inflammation and both reduced anxiety and increased exploration. We also observed a positive correlation between IFN-γ and the time spent in the guinea pig compartment but not the bobcat or fox compartments (Figure 5D), possibly reflecting different individual odor preferences in infected mice.

These results show that the cytokines present in the plasma, as part of the anti-parasitic immune response, tend to reflect the cyst burden in the CNS. In accordance with our previous observations (Figure 3G), high levels of pro-inflammatory cytokines are indicative of pronounced behavioral symptoms.

### ASP5 and MYR1 Distinctly Contribute to the Host Transcriptome Modulation

The strategies used by *T. gondii* to balance an excessive virulence and a poor replication, both undesirable outcomes for the parasite, include the secretion of modulators of the immune defense mechanisms in the host cell, in particular members of the dense granule family (GRA) (Bougdour et al., 2013, Braun et al., 2013, Braun et al., 2019, Gay et al., 2016, Olias et al., 2016). Prior to export, most GRA members undergo a proteolytic cleavage by ASP5 in the Golgi apparatus of the parasite (Coffey et al., 2015, Curt-Varesano et al., 2016, Hammoudi et al., 2015) and rely on the MYR1-dependent translocation machinery to cross the PVM (Franco et al., 2016) (Figure 3A). The PCA analyses on host gene expression data suggested specific differences in the host brain transcriptome when protein export was impaired (Figures 4A and 4B). To evaluate the effect of protein export on the host transcriptome, we compared differentially expressed genes in brains infected by either *T. gondii* ASP5-KO or MYR1-KO with *T. gondii* ME49-infected brains that had a comparable cyst load (∼200 cysts, referred to as ME49^low^). Host gene expression alterations resulting from ME49 infection were closer to those found with the MYR1-KO strain than with the ASP5-KO strain, suggesting a critical role played by ASP5 in modulating host gene expression (Figures S4A and S4B). However, pathway enrichment analysis on genes that were found to be upregulated in the ME49^low^-infected brains compared to the two mutant strains revealed that in both cases a large number of signaling pathways related to immunity were enriched (Figures S4C–S4E). Thus, when protein export is impaired, the inflammatory response in the host brain appears to be decreased.

Naturally, because animals infected with secretion mutants are characterized by a decreased cyst load, and behavioral alterations are minimal in mice with low cyst burden, we cannot separate the cyst number or inflammation from a potentially direct role played by the secreted proteins and, thus, determine how the export of effector proteins may modulate behavior.

### Cartography of Cyst Distribution in the Brain

**Figure 6:**
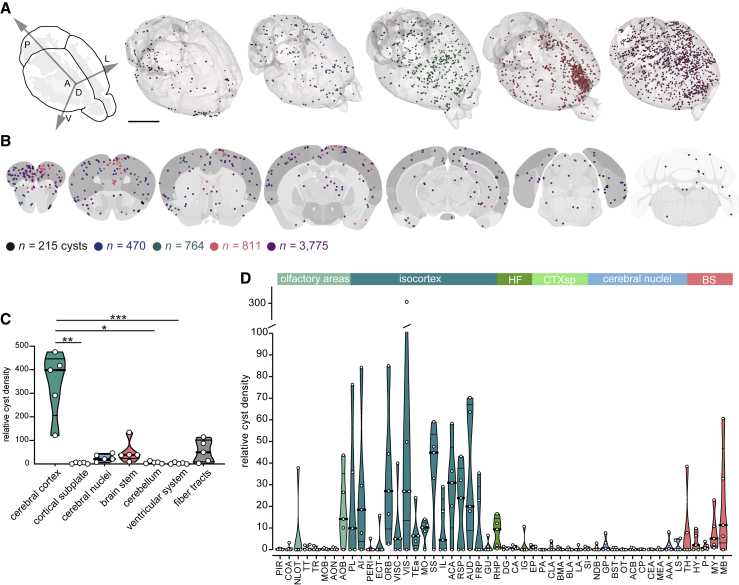
Cartography of *T. gondii* Cysts in the Mouse Brain (A) 3D rendering of CLARITY-processed brains showing colorized *T. gondii* ME49-GFP cysts. The size of the cysts was purposely made uniform. Scale bar, 5 mm. (B) Schematic coronal sections depicting detected *T. gondii* ME49-GFP cysts. One dot represents one cyst. Each depicted section holds cysts from 10 brain slices (spanning 50 μm). Numbers represent total number of detected and localized ME49-GFP cysts in the respective animals. Each individual is represented by a different color. (C) Relative cyst density (percentage of cysts in a region/volume of the region (mm^3^)) in different regions of the brains of mice infected with *T. gondii* ME49-GFP. (D) Distribution of the relative cyst density within subregions of the CNS of mice infected with *T. gondii* ME49-GFP. For abbreviations, see Table S5. For details on cyst counts, see Table S6. Bold bars indicate the median and thinner bars the quartiles. Each dot represents an individual.

To explore whether *T. gondii* displays a tropism bias, we evaluated the localization of cysts at the whole-brain scale with a high spatial resolution by using a home-built mesoscale light-sheet microscope (mesoSPIM) (Voigt et al., 2019). Five mice were infected either with ME49- and five ASP5-KO *T. gondii* strains expressing GFP under the control of a bradyzoite-specific promoter, and the corresponding brains were analyzed at 10 to 12 weeks p.i. An unbiased quantification of the cyst number based on the fluorescence signal revealed a wide inter-individual variation in cyst burden (Figure S6A), within the same range as observed with standard cyst quantification methods (Figure 3E). Volumetric analysis of the detected cysts indicated the co-existence of ME49 cysts of various sizes, suggesting an alternation of growth and rupture phases underlying the renewal of the cyst population (Figure S6A). In ASP5-KO-infected animals, a predominance of small-sized cysts was observed (Figure S6A). A 3D representation of the five mice infected with *T. gondii* ME49 parasites revealed non-uniform cyst-distribution patterns between individuals and between brain regions (Figure 6A; Video S1). Visualization of cysts from all ME49-infected mice along the coronal plane indicated an enrichment of cysts in the frontal cortex, often in small clusters and possibly in proximity to large blood vessels (Konradt et al., 2016) (Figure 6B). To determine if cysts were significantly enriched in specific brain regions, we performed a registration of the imaged brains to the Allen Brain atlas, attributing cysts to more than 550 different subregions. In a first coarse division, cysts appeared enriched in the cerebral cortex (Figure 6C), a distribution that was confirmed when looking closer at specific subregions and expanded to olfactory areas and brain stem structures (Figure 6D). Cortical subregions with the highest median cyst density were the somatosensory areas (SSs), the anterior cingulate area (ACA), the orbital area (ORB), the visual areas (VIS), the retrosplenial area (RSP), and the frontal pole (FRP) (Figures 6D and S6B). In ASP5-KO-infected mice, where cyst burden is very low, the distribution of cysts showed higher variability (Figures S6C–S6F), with most regions devoid of cysts, except for a few small clusters.

Video S1. 3D Rendering of CLARITY-Processed Brains Showing Colorized *T. gondii* ME49-GFP Cysts, Related to Figure 6

Thus, despite enrichment in cortical areas and the brain stem, we observed heterogeneity in cyst number and distribution between different individuals, suggesting a random infection and dissemination process, possibly through the anterior cerebral vascular system.

## Discussion

Infection by *T. gondii* can drastically affect the behavior of the intermediate host, notably the innate fear of felids in rodents. The mechanisms underlying the changes in valence of felid cues in infected mice are still unknown. Through a thorough behavioral analysis of *T. gondii*-infected mice, we demonstrate that *T. gondii* chronic infection triggers a decrease in anxiety, an increase in exploratory behaviors, and a general loss of aversion to predators, with no specificity for felids. Furthermore, our results point toward unspecific and immune-related changes in the brains of infected mice associated with altered behaviors. These findings contrast with previous reports supporting a highly specific attraction toward felid odors displayed by *T. gondii*-infected rodents (Lamberton et al., 2008, Vyas et al., 2007).

### Behavioral Alterations Caused by *Toxoplasma gondii* Are Associated with CNS Inflammation

The direct effects of *T. gondii* on neuronal integrity as well as the indirect changes induced through the activation of the immune system have been largely described (Carruthers and Suzuki, 2007, Dupont et al., 2012, Klein et al., 2017, Tedford and McConkey, 2017, Tyebji et al., 2019). However, few direct connections between these alterations and their potential role in the behavioral manipulation have been established. Here, we show that *T. gondii* ME49-infected mice exhibit sustained inflammation not only during acute infection but also during chronic infection. Thus, after clearance of circulating parasites from the mouse organism 2 or 3 weeks p.i., several inflammation markers are kept upregulated both at the transcriptomic and at the protein levels. Moreover, and importantly, both gene expression levels and concentration of plasmatic pro-inflammatory cytokines vary according to the cyst load, and all three parameters correlate with the severity of a behavioral alteration characteristic of *T. gondii* infection. This finding expands on previous associations between cyst or parasite load and certain aspects of behavior (Afonso et al., 2012, Evans et al., 2014, Hermes et al., 2008, Xiao et al., 2016) and points to a striking association between the transcription level of specific genes and altered behaviors.

Given the broad range of behavioral phenotypes that we and others observed in infected mice (such as alterations of motor and learning performance, memory, sociability, dominance, mate choice, anxiety, locomotor activity, and exploration) (Worth et al., 2014), it is conceivable that these reflect side effects of neuroinflammation, rather than result from a targeted modification of a specific neural circuit. We show that numerous transcriptional and neuronal alterations are tightly linked to cyst load. Among others, we observed a link between cyst burden and markers associated with neuronal loss, an upregulation of apoptotic and excitotoxic pathways, astrocyte activation, pro- and anti-inflammatory cascade, as well as a downregulation of neurotransmitter pathways. Taken together, our data suggest that the neuro-immune response plays a major role in the modulation of host behavior. Whether sustained inflammation is required for the behavioral alteration and whether the infection with *T. gondii* differs from other neurotropic infections such as meningitis remain open questions.

### Parasite Effector Proteins Contribute to the Host Transcriptome Remodeling during Persistence

Our analysis of the host transcriptome during chronic toxoplasmosis relative to other published reports showed a large overlap in the identity of differentially expressed genes, regardless of the timing in the chronic phase, the sex of the animals, or the mouse or parasite strain (Garfoot et al., 2019, Pittman et al., 2014, Tanaka et al., 2013). Immune-related genes were highly expressed in the CNS during *T. gondii* persistence, suggesting a continuous need to control the infection, which is maintained because of rupturing cysts. The overexpression of immune-related genes is likely to result from the massive infiltration of immune cells in the CNS of infected mice (Dupont et al., 2012). It is less clear whether the downregulation of specific genes results from a change in cell composition in the CNS or from transcriptional changes at the cellular level.

The inflammatory response was minimal when protein export from the parasite to the host cell was impaired. In the absence of ASP5, the expression of host immune-related genes varies minimally, whereas the deletion of MYR1 caused changes similar to those observed after infection with a wild-type strain. This observation is meaningful as MYR1 is a substrate of ASP5, a protease that also impacts on PVM resident proteins and the intravacuolar tubulovesicular network that are MYR1 independent. Overall, these data highlight the important contribution of *T. gondii* effector proteins to the remodeling of the host transcriptome during the chronic stage.

### Tissue-Cyst Distribution Is Widespread in the CNS

To map the precise location and number of cysts in the brains of *T. gondii*-infected mice, we developed a novel tool using light-sheet microscopy. We found that the cyst density was highest in the cortical plate but with inter-individual variability regarding which specific subregions were most infected. The absence of a specific tropism for brain regions involved in predator aversion and other altered behaviors point toward a behavioral manipulation mediated by neuronal inflammation rather than by direct interference of the parasite itself with specific neuronal populations. In line with our findings, a recent study suggests that the determinant factor for *T. gondii*-induced hyperactivity is neuroinflammation, independent of cyst load (Martynowicz et al., 2019). Also, cysts do not necessarily colocalize with inflammatory centers and neural lesions (Hermes et al., 2008, Martynowicz et al., 2019, Parlog et al., 2014, Torres et al., 2018), and whether their persistance is necessary for the behavioral manipulation of the host is not clear (Ingram et al., 2013, Martynowicz et al., 2019).

In accordance with this assumption, the parasite *Eimeria vermiformis* is causing a reduced avoidance of predator in infected mice, but contrary to *T. gondii*, *E. vermiformis* is monoxenous, as it has a direct life cycle involving no intermediate hosts, and it is not a neurotropic parasite with the whole replication process taking place in epithelial cell of the gastrointestinal tract (Kavaliers and Colwell, 1995).

### Toxoplasmosis in Mice versus Humans: Distinct Pathogenesis and Variable Behavioral Consequences

Humans generally exhibit less symptoms than rodents following *T. gondii* infection. The pathogenicity, inflammatory response, and parasite persistence are indeed different between the two species. Our study shows a general loss of inhibition in infected rodents and the severity of these symptoms is correlated with the cyst load and inflammation levels. In humans, multiple studies have shown an association of *T. gondii* infection and, among others, an increased risk of schizophrenia, traffic accidents, and suicide attempts (Burgdorf et al., 2019, Sugden et al., 2016, Sutterland et al., 2019, Xiao et al., 2018). Although we should be very cautious in making direct translations of our findings based on mouse behavior to human behaviors and psychology, it is clear that inflammatory responses in the CNS are known to contribute to neuronal damage associated with neurodegenerative diseases and may mediate Alzheimer’s disease pathogenesis (Block et al., 2007, Heppner et al., 2015, Klein et al., 2017). Thus, the potential effect of *T. gondii* infection on neuronal function in humans should not be underestimated.

### *Toxoplasma gondii* Exerts a Non-specific but Adaptive Manipulation on Rodent Intermediate Hosts

Aspecific behavioral manipulation of intermediate hosts by *T. gondii* has been previously suggested (Worth et al., 2013, Worth et al., 2014). In the current study, we show, based on multimodal experimental data, that the feline-specific fatal attraction theory is flawed. So why is the idea of a specific manipulation by *T. gondii* in rodents still alive in the field and outside the scientific community? Possibly, the specificity of a behavioral hijack is an exciting idea that pleases both biologists and the public, a situation that may have somehow helped to nourish a myth. One of the most attractive aspects of the specific feline attraction theory is that it represents an adaptive manipulation of a mammal by a parasite. One could argue that the non-specificity we show here could weaken this view. But, in fact, non-specific manipulations can be adaptive, depending on the initial predation risk of the intermediate host (Seppälä et al., 2008). A lower general anxiety in infected rodents and other intermediate hosts would not only facilitate the capture of infected prey by felids but also scavenging by other predators favoring the non-sexual transmission between intermediate hosts and, thus, explain the successful spread of the *Toxoplasma* population and its propensity to be clonal.

## STAR★Methods

### Key Resources Table

**Table:** 

REAGENT or RESOURCE	SOURCE	IDENTIFIER
**Bacterial and Virus Strains**		

*E. coli* XL-10 Gold	Strategene	Cat# 200314

**Chemicals, Peptides, and Recombinant Proteins**		

Pentobarbital Esconarkon	Streuli Pharma	Cat# QN51AA01
Odor stimulus: bobcat urine	Main Outdoor Solutions	Cat# 91012
Odor stimulus: fox urine	Main Outdoor Solutions	Cat# 91412

**Critical Commercial Assays**		

ACTH ELISA kit	MD Bioproducts	Cat# M046006
Corticosterone ELISA kit	Enzo Life Sciences	Cat# ADI-900-097
Mouse IL-12/IL-23 total p40 Uncoated ELISA	Invitrogen	Cat# 88-7120
Mouse IFN gamma Uncoated ELISA		Cat# 88-7314
**Deposited Data**		

Raw fastq RNA-seq files	This paper	PRJEB32699

**Experimental Models: Cell Lines**		

Human foreskin fibroblasts (HFFs)	ATCC	CRL-2429

**Experimental Models: Organisms/Strains**		

Mouse: B6CBAF1/JRj	Janvier Labs	N/A
*T. gondii*: ME49	ATCC	506111
*T. gondii*: ME49Δ*hxgprt* pBAG1-GFP	This study	N/A
*T. gondii*: ME49Δ*asp5*	Hammoudi et al., 2015	N/A
*T. gondii*: ME49Δ*asp5*Δ*hxgprt* pBAG1-GFP	This study	N/A
*T. gondii*: ME49Δ*myr1*	This study	N/A
*T. gondii*: ME49Δ*myoJ*	Frénal et al. 2017	N/A
*N. caninum*: Nc-Spain 7	Arranz-Solís et al., 2015	N/A

**Software and Algorithms**		

SMART videotracking software v3.0	Panlab	Cat# 76-0695
GraphPad Prism 8	GraphPad Software	https://www.graphpad.com/scientific-software/prism/
FASTX-Toolkit	Hannon Lab	http://hannonlab.cshl.edu/fastx_toolkit/
Trim Galore v0.4.2	Babraham Bioinformatics	http://www.bioinformatics.babraham.ac.uk/projects/trim_galore/
HISAT2 v2.1.0	Kim et al., 2015	https://ccb.jhu.edu/software/hisat2/index.shtml
SAMtools v1.5	Li et al., 2009	http://samtools.sourceforge.net/
HTSeq-count v0.9.1	Anders et al., 2014	https://htseq.readthedocs.io/en/release_0.11.1/count.html
edgeR v3.24.3	Robinson et al., 2010	https://bioconductor.org/packages/release/bioc/html/edgeR.html
pca3d v0.1	Weiner et al., 2012	https://cran.r-project.org/web/packages/pca3d/index.html
ggplot2 v3.1.1	Wickham, 2016	https://ggplot2.tidyverse.org/reference/index.html
gplots v3.0.1.1	Warnes, 2011	https://cran.r-project.org/web/packages/gplots/index.html
GOplot v1.0.2	Walter et al., 2015	https://wencke.github.io/
RStudio v1.1.463	R Studio Team, 2015	https://rstudio.com/
Mousemine: GeneID information, Pathway and GO enrichment analysis	Motenko et al., 2015	http://www.mousemine.org/mousemine/begin.do
Amira v 2019.1	Thermo Fisher Scientific	https://www.fei.com/software/amira/
Imaris v 9.3.0	Oxford Intruments	https://imaris.oxinst.com/

**Other**		

X-CLARITY protocol	Chung lab	http://www.chunglabresources.com/cl1#cl-protocol
mesoSPIM Initiative		http://mesoSPIM.org

### Lead Contact and Materials Availability

Further information and requests for resources and reagents should be directed to and will be fulfilled by the Lead Contact, Dominique Soldati-Favre (Dominique.Soldati-Favre@unige.ch). All unique/stable reagents generated in this study are available from the Lead Contact without restriction.

### Experimental Model and Subject Details

#### Ethics statements

All animal experiments were carried out with the authorization GE/138/17 according to the guidelines and regulations issues by the Swiss Federal Veterinary Office.

#### Animals

Experiments reported here involved male B6CBAF1/J mice all purchased at 5 weeks of age from Janvier Laboratories, France. Animals were group-housed (3-6 animals/cage) at 20°C under a 12 hours light/dark cycle with *ad libitum* access to food and water. Except for *T. gondii* infection, animals were specific-pathogen free. Animals were infected at 6-7 weeks old. All behavioral tests were conducted at 4-8 weeks post infection, when animals were 10-15 weeks old. To ensure that animals were naive to stimulus odors they were only used for a single behavioral trial. Mice were sacrificed between 7 to 8 weeks post infection by i.p. injection with a lethal dose of pentobarbital (150 mg/kg). Stimulus animals used in the interaction test were adult male BALB/c mice. Stimulus mice used in the sociability test were adult male CBA mice. Stimulus rats were 8 weeks old male Lewis rats. Guinea pigs used as urine donors were adult Hartley females.

#### Bacteria, parasite and host cell culture

*E. coli* XL-10 Gold chemo-competent bacteria were used for all recombinant DNA experiments. *T. gondii* (type II ME49 strain obtained from ATCC, numbers 50611) and *N. caninum* (Nc-Spain7) (Arranz-Solís et al., 2015) tachyzoites parental and derivative strains were grown in confluent human foreskin fibroblasts (HFFs) maintained in Dulbecco’s Modified Eagle’s Medium (DMEM, GIBCO) supplemented with 5% fetal calf serum (FCS), 2 mM glutamine and 25 mg/ml and 25 mg/ml gentamicin.

#### DNA vector construct

To generate the ME49Δ*hxgprt* P_BAG1_-GFP strain, the *hxgprt* locus (TGME49_200320) has been disrupted into the type II ME49 strain using a Cas9.YFP/CRISPR gRNA targeting the *hxgprt* coding sequence. The gRNA3 was generated using the Q5 site directed mutagenesis kit (NEB) with the primer pair gRNA3/gRNA-4883 and using the vector pSAG1::CAS9-GFP-U6::sgUPRT as template (Shen et al., 2014). In this background, the P_BAG1_-GFP-HXGPRT construct was stably integrated in the genome after *Not*I linearization.

To generate the ME49Δ*asp5*Δ*hxgprt* P_BAG1_-GFP strain, the *hxgprt* locus has been disrupted into the type II ME49Δ*asp5* strain (Hammoudi et al., 2015) using the gRNA3. In this background, the P_BAG1_-GFP-HXGPRT construct was stably integrated in the genome after *Not*I linearization.

To generate the ME49Δ*myr1* strain, a PCR fragment encoding the DHFR-TS selection cassette was generated using the KOD DNA polymerase with the vector p2854-DHFR (Donald and Roos, 1993) as template and the primers P5/P6 that also carry 30 bp homology with the 5′ and 3′ ends of *myr1*. To direct the insertion of the PCR product, a specific dgRNA vector carrying gRNA1 and gRNA2 has been generated as previously described (Hammoudi et al., 2018). For primers used in this study, see Table S4.

#### Parasite transfection and selection of clonal stable lines

*T. gondii* tachyzoites were transfected by electroporation as previously described (Soldati and Boothroyd, 1993). Parasites disrupted for the *hxgprt* locus expressing Cas9-YFP were sorted by flow cytometry (FACS) and the clones were analyzed by sequencing (Figures S3A–S3C). Selection of transgenic parasites were performed either with mycophenolic acid and xanthine for HXGPRT selection (Donald et al., 1996) or pyrimethamine for DHFR selection (Donald and Roos, 1993). Stable line for all expressing strains were cloned by limited dilution and checked for genomic integration by PCR.

#### Preparation of T. gondii genomic DNA

Genomic DNA (gDNA) was prepared from tachyzoites of ME49Δ*hxgprt* P_BAG1_-GFP, ME49Δ*asp5*Δ*hxgprt* P_BAG1_-GFP or ME49Δ*myr1* strains using the Wizard SV genomic DNA purification (Promega) according to instruction of the manufacturer.

#### Mice infection

6-weeks old B6CBAF1/J male mice (Janvier laboratories) were infected with 2.10^2^ and, if specified, with 5.10^1^ or 10^3^ parasites. All infections were administered i.p. in 200 μL. Freshly egressed tachyzoites from HFFs monolayers were prepared immediately before mouse infection. The sham groups were injected with PBS.

### Method Details

#### Behavior studies

All behavioral tests, except the holeboard and the predator avoidance tests, took place during the light phase. Predator avoidance and holeboard tests started 1 hour after the onset of the dark phase and took place under dim red light. Behavior was recorded with a GoPro® camera placed on top of the arenas. All behaviors were analyzed with the SMART® software (PanLab). All behaviors were either analyzed automatically or by an observer blind to the conditions. All behavioral tests were performed in a neutral room within the animal facility. Behavioral tests were carried out with multiple batches of mice, where each batch always contained mice from the appropriate control groups. For all detailed results of behavioral assays, cyst counts and physiology, see Table S2.

#### Open field (OF)

The arena of the OF consisted in a square (40 × 40 × 20 cm) with walls made out of white synthetic cardboard and the floor covered with paper tablecloth that was changed between each mouse. The arena was homogenously lit (400 lux) and covered with a perforated Plexiglas lid. Mice were transferred to the behavior room where they were immediately placed in center of the arena and behavior was recorded for 5 min. The following parameters were analyzed: percentage of time spent in the center of the arena (20 × 20 cm), the total distance traveled, time spent rearing (standing on hind legs either freestanding or against the wall) and investigating (active olfactory investigation of the borders and corners of the arena).

#### Hand investigation (HI)

The hand of the experimenter, wearing a clean glove, was positioned on one side of a standard cage (32 × 17 × 14 cm) filled with clean bedding, the back of the fingers touching the floor and leaving just enough space for the mouse to pass around the hand. Mice were placed on the opposite side of the cage and their behavior was recorded for 5 min. The following parameters were analyzed: latency to the first touch of the hand (placing a paw on the hand), time spent on each half of the cage, time spent in contact with hand (either touching with forepaws, walking on hand, biting or close olfactory investigation), foraging and burying the hand with bedding, risk assessment behavior (approaching slowly the hand with a stretched body posture), and tail rattling.

#### Elevated plus-maze (EPM)

The elevated plus maze arena consisted in an elevated (41 cm) plus-shaped arena where two of the arms (65 × 6 cm) have walls (15 cm) and two of the arms are open. Mice were placed in the center of the plus, facing a closed arm. Behavior was recorded during 5 min. The following parameters were analyzed: percentage of time spent in open and closed arms respectively, the total distance traveled, percentage of distance in open arm and the number of visits to the end of the open arms (last 5 cm).

#### General exploration (EXP)

The exploration test took place in the open field arena (same as described above), where different stimuli were placed in the corners of the arena. The arena contained one food stimulus (apple), one neutral object (metallic cube with holes) and a social stimulus (unfamiliar male BALB/c mouse in a cylindrical cage). Mice were allowed to explore the objects for 10 min. The time spent investigating each stimulus was measured (nose within 0.5 cm from the stimulus or climbing on stimulus).

#### Holeboard (HB)

Exploration in the holeboard test was assessed as described by Crawley (1985). The holeboard arena consisted in a square arena (40 × 40 cm) with a floor plate raised 10 cm above the ground. The floor plate contained 16 holes of 3 cm diameter equally distributed in the central area (starting at 6 cm from the border). The walls of the arena were made of transparent Plexiglas. At the onset of the dark phase mice were transferred to the behavior room for 1 hour of habituation to the environment. Then, mice were placed in the arena and behavior was recorded for 10 minutes with cameras placed above and on the side of the arena. The number and the total time of exploratory behaviors (rearing and head dips) were quantified.

#### Novel object recognition (NOR)

The exploration test took place in the open field arena (same as described above), following a standard protocol (Leger et al., 2013). In the familiarization session mice were exposed to two identical objects (A and A’) for 10 minutes. Mice were put back into their home cages for one hour. In the test session, mice were exposed to one familiar object (A or A’) and one new object (B) for 5 min. Both objects used were on equal valence and were equally used as familiar or new objects. Behavior was recorded and investigation of the objects was measured manually (olfactory investigation and leaning on the objects). Climbing on the objects was not considered as investigation.

#### Bobcat aversion (BA)

Mice were habituated 3x and tested on day 4. For habituation, mice were transferred to the behavior room at the onset of the dark phase. They were placed for 10 min in a two-chamber arena with one large compartment (38.5 × 19.5 × 22 cm) with transparent Plexiglas walls (exposed compartment) and a smaller hideaway compartment (31.5 × 17.5 × 10 cm) with red transparent Plexiglas walls (appearing as dark to the mouse). Both compartments were interconnected by a door. On the test day, a Petri-dish containing bedding from the homecage of the mouse was placed inside the hideaway. Mice were first placed in the arena containing only their bedding for 5 min and their behavior was recorded (pre-exposure). Then they were confined to the hideaway and the door was closed. Another Petri-dish containing 1 mL of pure bobcat urine (Maine Outdoor Solutions) spotted on blotting paper was introduced in the open chamber. The door was opened and behavior was recorded for 10 minutes. The time spent in each compartment as well as the time spent investigating the odor source was quantified. The aversion index was calculated as follows: %time spent in open compartment with bobcat odor / %time spent in open compartment without odor.

#### Sociability

Protocol for sociability test was adapted from Yang et al. (2011). The test took place during the light phase but under dim lights (20 lux). Mice were transferred to the behavior room at least 1 hour before the start of the test for habituation to the environment. Mice were first introduced into an empty three-chamber arena (each chamber is 38.5 × 19.5 × 22 cm) interconnected by doors. Mice could explore the arena for 10 minutes. Behavior was recorded and time spent in each compartment was measured. Then the animal was confined to the center chamber, the doors were closed and stimuli were introduced into the side chambers. On one side an unfamiliar mouse (CBACaCrl adult male mouse, previously habituated to the test situation) inside a transparent Plexiglas cylinder that allows for visual and olfactory investigation and on the other side a non-social object (small colored glass bottle) inside a cylinder. Doors were opened and behavior was recorded for 10 min. The time spent in each compartment was measured.

#### Predator aversion (PA)

Mice were habituated on 3 days. For habituation, mice were transferred to the behavior room at the onset of the dark phase. The behavior room was lit with dim red light. After 1 hour of habituation to the room, they were placed for 10 min in a four-chamber arena. This arena (40 × 40 cm) consisted in a square divided into 4 chambers (19.5 × 19.5 cm), interconnected in the center. The walls of the arena were made out of transparent Plexiglas, the floor was covered with paper and the top was covered with perforated Plexiglas. The arena was placed within a larger square of white cardboard, avoiding any external visual stimuli to interfere with the behavior. On day 4, the following olfactory stimuli were placed in each chamber of the arena, in a pseudo-randomized order: 1 mL bobcat urine diluted 1:2 (Maine Outdoor Solutions), 1 mL of fox urine diluted 1:2 (Maine Outdoor Solutions), 1 mL of guinea pig urine diluted 1:2 (collected from guinea pigs in metabolic cages), and bedding from the mouse’s home cage. All stimuli were placed in Petri-dishes that were attached in the corners of the chambers using tape. Urinary stimuli were pipetted on two squares of blotting paper (2 × 2 cm) (ref). All Petri-dishes had a lid with a central opening (1.5 × 1.5 cm), allowing direct contact with the stimuli but preventing the mice from removing the blotting papers from the dish. Mice were placed in the chamber containing their own bedding, facing the Petri-dish. Their behavior was recorded for 10 min. The time spent investigating each odor (head over the Petri-dish) was measured.

#### Live predator avoidance (LPA)

The test took place in the same two-chamber arena as described for the bobcat aversion test. Mice were habituated once and tested on the next day. On the test day, mice were first placed in the empty arena for 5 min and their behavior was recorded (pre-exposure). Then they were confined to the hideaway and the door was closed. An anesthetized male rat (injected with 75 mg/kg of ketamine 100 and 10 mg/kg of xylazine 2%) was placed inside the exposed compartment. In order to maintain rat body temperature, the rat was placed on a foot warmer (Thermopad GmbH). The door was opened and behavior was recorded for 10 min. The time spent in each compartment was measured.

#### ELISA

Mice were euthanized with a 150 mg/kg Pentobarbital (Esconarkon, Streuli Pharma) injection before decapitation. Trunk blood was collected in tubes containing 100 μl of 0.5 M EDTA on ice. Blood was centrifuged at 2000 g for 4 min at 4°C. Plasma was collected and stored at −20°C. Plasma ACTH and corticosterone concentration was measured by ELISA (stress hormone ELISA kit, MDBiosciences; Corticosterone ELISA kit, Enzo Life Sciences; IFN-γ and IL-12/IL-23 ELISA kits, Invitrogen) according to the manufacturer’s protocols. Absorbance was read at 450 and 405 nm with a microplate reader (Viktor x5, Perkin Elmer).

#### Brain processing and tissue cysts counting

Mice brains were homogenized in 1 mL PBS and syringe passaged 5-10 times through an 18G needle to break up large clumps. Then, the homogenate was sequentially syringe passaged through a 20G needle and a 23G needle (10 times each). Tissue cysts number was estimated by counting 5 fractions of 10 μl from each brain homogenate using the 10 × and 20 × objectives of an inverted microscope.

#### RNA isolation and Sequencing

RNA was isolated employing a hybrid RNA extraction protocol with TRIzol (Life Technologies) and QIAGEN RNeasy Mini Kit. 100uL of homogenized brain was lysed with 1 mL TRIzol and centrifuged to remove debris, following which chloroform was added to separate the aqueous layer and the organic layer. The upper phase which contains RNA was precipitated with 70% ethanol and is further processed using the the RNeasy column according to the manufacturer’s instructions. The quantity and quality of the extracted RNA was measured with a Qubit Fluorometer (Life Technologies) and an Agilent 2100 BioAnalyser (Agilent Technologies) respectively. Ribosomal RNA was removed by applying poly A selection. RNA was subjected to 100 bp single read sequencing on a Illumina HiSeq 4000 (Illumina, San Diego, CA, USA) at the iGE3 Genomics platform at the University of Geneva (https://ige3.genomics.unige.ch). Samples Uninfected (U7, U8, U17, U18) and ME49-infected (T8, T19, T20, T28, T26, T27) were processed together and multiplexed in one sequencing lane of the flow cell. Samples Uninfected (U25, U26), ME49-infected (T31, T36, T37), ASP5-KO-infected (A1, A3, A4) and MYR1-KO (M2, M12, M3) were processed together and multiplexed in one sequencing lane of the flow cell. Raw reads generated in the current study were deposited at the following link and accession number at the European Nucleotide Archive (https://www.ebi.ac.uk/ena/) PRJEB32699.

#### RNaseq data processing and analysis

The quality of the reads was assessed with FASTX-Toolkit (v0.0.13). The Illumina adaptor sequences from the raw reads and the poor quality reads were removed from the dataset (parameters –q 30 and–length 36) with Trim Galore v 0.4.2. The resulting curated reads were aligned to the mouse reference genome (GRCm38/mm10) using the HISAT2 (v2.1.0) aligner. Read counts on the genome features were generated using HTSeq-count (v0.9.1). The raw counts of the samples are reported in Table S3. The high performance computing Baobab cluster at University of Geneva was utilized to perform all the computations.

Differences in gene expression was assessed using edgeR (v3.24.3), a Bioconductor package in R). Only genes with > 1 counts per million across at least one of the conditions were considered. As calculated by exactTest with default settings for differences between the two groups of negative binomial counts, only genes with at least a logFC of ± 1 and an adjusted p value (FDR) less than 0.05 were treated as differentially expressed. Normalized expression values from the count data for plotting and clustering were obtained from the normalization factors calculated by the TMM (trimmed mean of the Mvalues) method (Table S3). Principal Component Analysis (PCA) was performed on scaled normalized expression values using the built-in R function prcomp(), and the plots were generated using ggplot2 (v3.1.1) and pca3d (v0.1) R packages. The heatmaps for the genes of interest were also generated in R using the heatmap.2 function of the gplots (v3.0.1.1) R package. Bubble plots for pathway enrichment visualization were generated using the GOplot (v1.0.2) package. All R analysis was done using RStudio environment (v1.1.463). Mousemine was used for Pathways (data from Reactome) and GO enrichment analysis of selected gene sets, as described in the Results section, using Benjamini-Hochberg test correction (p < 0.05). The GeneIDs of the various lists are listed in Table S3.

#### CLARITY and 3D image acquisition

Mice were perfused with 4% PFA and tissue was postfixed overnight in 4% PFA. Brains were clarified following the CLARITY protocol (Chung et al., 2013), using X-CLARITY, an electrophoretic tissue clearing commercial system (https://logosbio.com). Briefly, brains were infused in a hydrogel-based solution. Hydrogel is a monomer polymerizable upon heating which binds to the N-terminal of proteins but not to the lipids. Heating the brain at 37°C for 3 hours triggers the hybridization of tissue with polymer and the formation of a mesh on which all proteins are attached. A final electrophoresis in a detergent medium (Sodium Dodecyl Sulfate - SDS) leads to the removal of lipids from the brain. Finally, brains were immersed in a refractive index matching solution (RIMS) containing Histodenz (Sigma Aldrich) for at least 24 hours before being imaged.

After clearing, brains were glued to a holder and immersed in a 10 × 20 × 45 mm quartz cuvette filled with RIMS. The cuvette is then placed in a chamber filled with oil with n_D_ = 1.45 (Cargill). Imaging is performed with a home-built mesoscale single-plane illumination microscope. The microscope consists of a dual-sided excitation path using a fiber-coupled multiline laser combiner (405, 488, 561 and 647 nm, Toptica MLE) and a detection path comprising an Olympus MVX-10 zoom macroscope with a 1 × objective (Olympus MVPLAPO 1 × ), a filter wheel (Ludl 96A350), and a scientific CMOS (sCMOS) camera (Hamamatsu Orca Flash 4.0 V3). The excitation paths also contain galvo scanners for light-sheet generation and reduction of shadow artifacts due to absorption of the light-sheet. In addition, the beam waist is scanned using electrically tunable lenses (ETL, Optotune EL-16-40-5D-TC-L) synchronized with the rolling shutter of the sCMOS camera. This axially scanned light-sheet mode (ASLM) leads to an uniform axial resolution across the field-of-view (FOV) of 5 μm. Image acquisition is done using custom software written in Python. Z stacks were acquired at 5 μm spacing with a zoom set at 0.8x resulting in an *in-plane* spatial resolution of 7.8 μm (2048x2048 pixels). Excitation wavelength of the cyst was set at 488 nm with an emission filter 530/40 nm bandpass filter (BrightLine HC, AHF). Autofluorescence excited at 561 nm and detected with a LP 561 was used for registration. Complete description of the mesoSPIM microscope is available here (Voigt et al., 2019).

#### Brain images processing

Z stacks were processed using Amira (Thermo Fisher Scientific) to segment the brain and the cysts. Videos showing 3D reconstituted *T. gondii*-infected brains were generated using Imaris (Oxford Instruments).

#### Registration to the Allen Reference Atlas

To perform accurate mapping between CLARITY and the Allen Reference Atlas (ARA), we developed specialized workflows optimized for multi-modal registration of clarified data, based on tools from ANTs (Avants et al., 2011). The workflows included pre-processing, brain extraction, intensity correction, orientation estimation, registration initialization, as well as optimized similarity metrics, optimization and regularization parameters for the multi-stage, multi-resolution registrations. Image registration between the autofluorescence CLARITY volume and ARA template relied on intensity-based alignment. The ARA 10 μm template was chosen as the reference image for the registration steps and the ARA 10 μm labels were subsequently warped and up-sampled to CLARITY full-resolution, native space. The registration steps consisted of an intensity-based b-spline, three-stage registration with increasing degrees of freedom of their transformations, encompassing a) a rigid 6 degrees of freedom (DOF), b) an affine (12 DOF), and c) a non-rigid (deformable) b-spline symmetric normalization (SyN) stage, each consisting of a multi-resolution approach with 4 levels. We employed the mutual information (MI) similarity metric for the rigid and affine stages and cross correlation (CC) for the deformable stage (Avants et al., 2008). The net product of the registration is a transformation that performs bidirectional warping of images to and from the CLARITY native space, to and from ARA templates and labels.

### Quantification and Statistical Analysis

Results are always presented as mean ± SEM, unless something else is stated. For comparisons of behavioral parameters, cyst load and ELISA measures we first tested the normality of distribution with a D’Agostino & Pearson normality test. For comparisons of two independent normal distributions, we used a non-directional t test. For non-parametric data, we used a Mann-Whitney U test. For multiple comparisons of two groups, we used Two-way ANOVA followed by Sidak’s multiple comparisons test. For comparisons of more than two normal distributions, we used One-way ANOVAs followed by Dunnett’s multiple comparisons test. For comparisons of more than two non-parametric distributions, we used a Kruskal-Wallis test followed by Dunn’s multiple comparisons test. For repeated-measures in more than two non-parametric distributions, we used Friedmans test followed by Dunn’s multiple comparisons test. Correlations between two independent non-parametric distributions were measured using a Spearman correlation test. The details of all statistical analysis (number of samples analyzed, statistical test used, exact statistical values obtained) can be found in the Table S1. All analyzes were performed using Graphpad Prism 8. Statistcal analyses on data obtained in the RNaseq experiment are described separately in the STAR Methods section on RNaseq data processing and analysis above.

## Supplemental Information

Document S1. Figures S1–S6 and Tables S4 and S5

Table S1. Statistics, Related to Figures 1, 2, 3, 5, 6, S1, S3, S4, S5, and S6

Table S2. Behavioral and Physiology Data, Related to Figures 1, 2, 3, 4, 5, S1, S2, S3, S4, S5, and S6

Table S3. RNA Sequencing (RNA-Seq) Data, Related to Figures 4, 5, S4, and S5 The raw reads were deposited at the following link and accession number at the European Nucleotide Archive (https://www.ebi.ac.uk/ena/) PRJEB32699.

Table S6. Light-Sheet Data, Cyst Counts, Related to Figures 6 and S6

Document S2. Article plus Supplemental Information
