# Supplementary material for: Neuroinflammation-Associated Aspecific Manipulation of Mouse Predator Fear by Toxoplasma gondii
Source: Cell Rep. 2020 Jan 14;30(2):320–334.e6. doi: 10.1016/j.celrep.2019.12.019 (PMC6963786; doi:10.1016/j.celrep.2019.12.019)
Supplement: Document S1. Figures S1–S6 and Tables S4 and S5 [file mmc1.pdf]

Cell Reports, Volume *30*

Supplemental Information

Neuroinflammation-Associated Aspecific Manipulation of Mouse Predator Fear by *Toxoplasma gondii*

Madlaina Boillat, Pierre-Mehdi Hammoudi, Sunil Kumar Dogga, Stéphane Pagès, Maged Goubran, Ivan Rodriguez, and Dominique Soldati-Favre

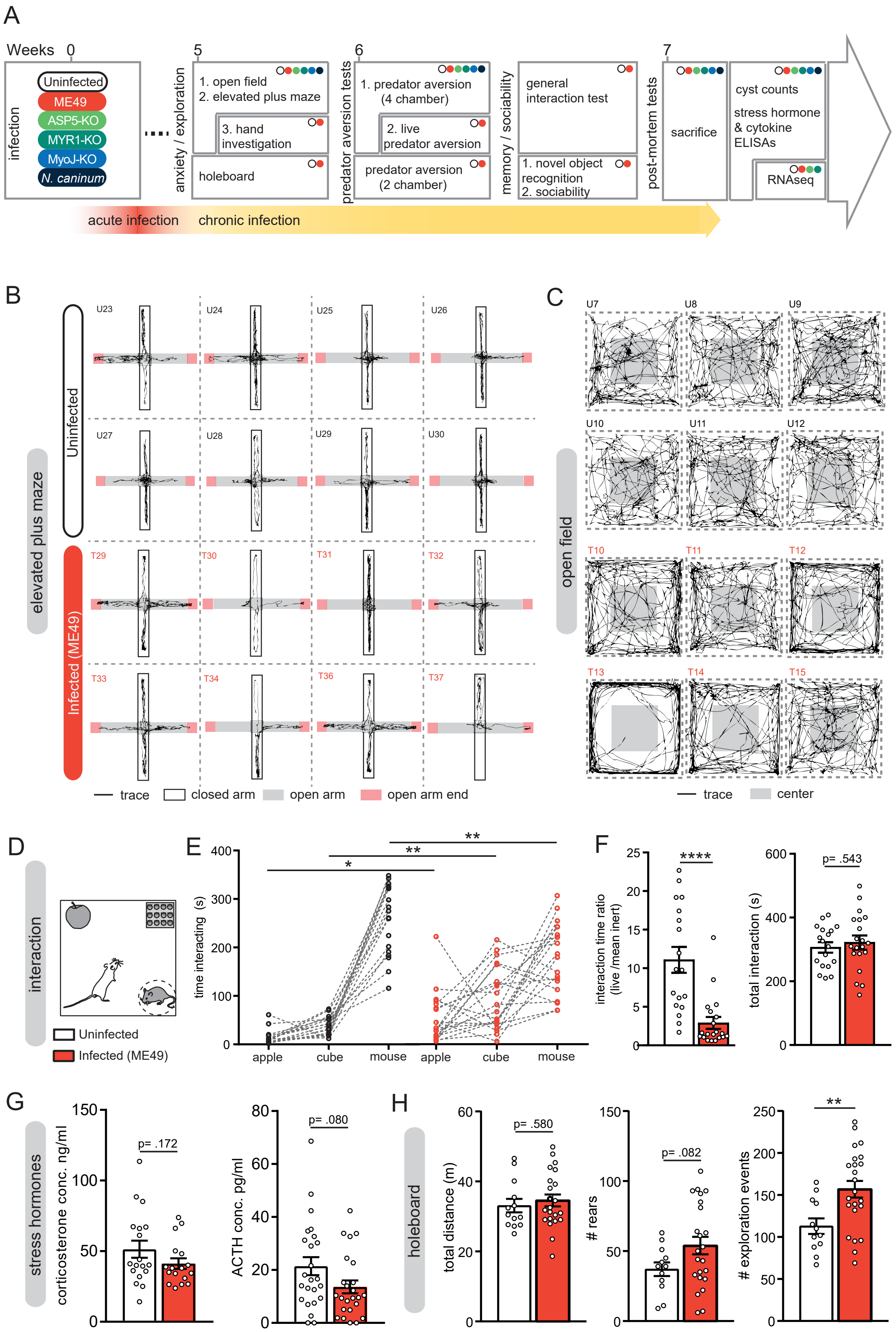

A
Weeks 0
infection
Uninfected
ME49
ASP5-KO
MYR1-KO
MyoJ-KO
N. caninum
5
anxiety / exploration
1. open field
2. elevated plus maze
3. hand investigation
holeboard
6
predator aversion tests
1. predator aversion (4 chamber)
2. live predator aversion
predator aversion (2 chamber)
memory / sociability
general interaction test
1. novel object recognition
2. sociability
7
post-mortem tests
sacrifice
cyst counts
stress hormone & cytokine ELISAs
RNAseq
acute infection
chronic infection
B
elevated plus maze
Uninfected
Infected (ME49)
U23 U24 U25 U26
U27 U28 U29 U30
T29 T30 T31 T32
T33 T34 T36 T37
trace
closed arm
open arm
open arm end
C
open field
U7 U8 U9
U10 U11 U12
T10 T11 T12
T13 T14 T15
trace
center
D
interaction
Uninfected
Infected (ME49)
E
time interacting (s)
apple cube mouse apple cube mouse
F
interaction time ratio (live /mean inert)
****
total interaction (s)
p= .543
G
stress hormones
corticosterone conc. ng/ml
p= .172
ACTH conc. pg/ml
p= .080
H
holeboard
total distance (m)
p= .580
# rears
p= .082
# exploration events
**

Figure S1 - *T. gondii* infection leads to decreased anxiety and increased exploration behaviors, Related to Figure 1

(A) Schematic of workflow and time course of infection. Dots in upper right corners describe which groups of mice were analyzed or performed the corresponding test. Precise details of experimental groups and all results can be found in Table S2.

(B) and (C) Representative traces from mice in the elevated plus maze and open field respectively. Numbers represent mice IDs. Traces only represent trajectory but not time spent in specific areas.

(D) Schematic of the general interaction test.

(E) Quantification of interaction times with each stimulus and (F) ratio of time spent interacting with the live stimulus (mouse) / mean inert stimuli (apple + cube).

(G) Basal corticosterone plasma levels (uninfected n=17, infected (ME49) n=18) and basal adrenocorticotropic hormone (ACTH) plasma levels (uninfected n=24, infected (ME49) n=24) of mice at 7 weeks post-infection.

(H) Quantification of behaviors during the holeboard test. Exploration events comprise head dips and rears.

Bars indicate mean ± SEM and each dot represents an individual. For details of the statistical analyses, see Table S1.

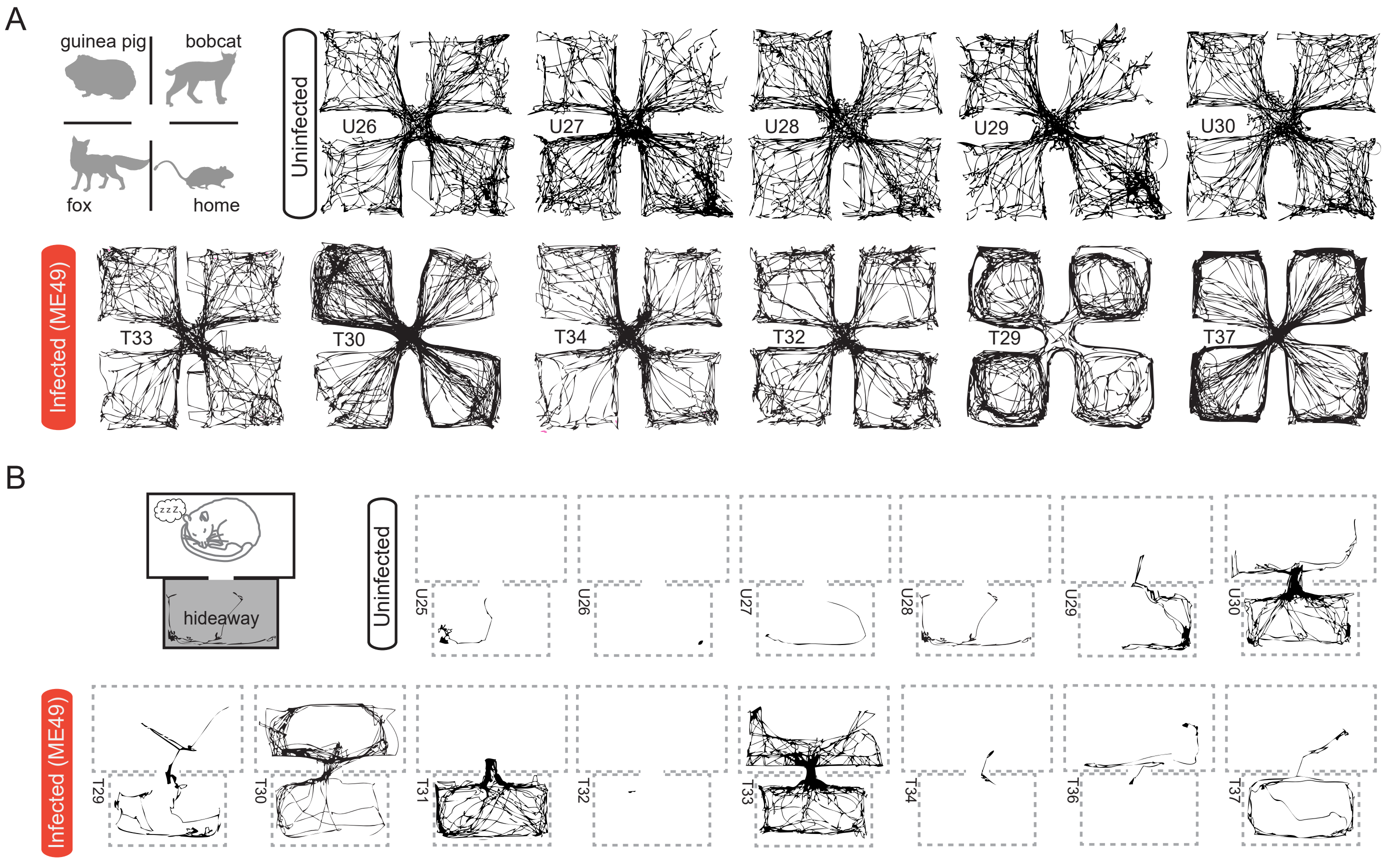

Figure S2 – Non-specific loss of predator aversion in *T. gondii* infected mice, Related to Figure 2
(A) Representative traces from uninfected and *T. gondii* ME49-infected mice in the 4-chamber predator aversion assay. Numbers indicate mice IDs.
(B) Representative traces from uninfected and *T. gondii* ME49-infected mice in the live predator aversion assay.

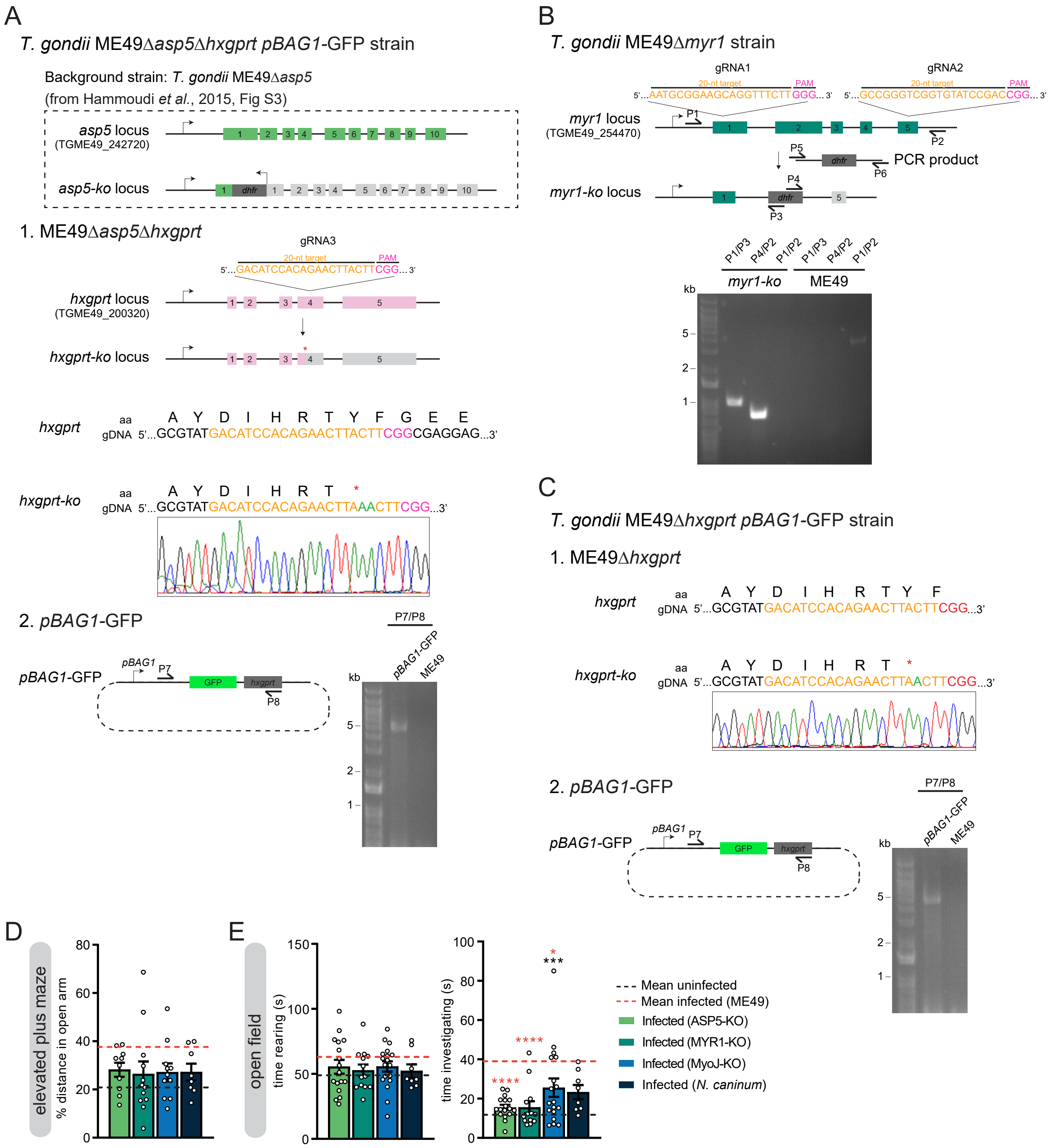

Figure S3 - Severity of behavioral changes induced by *T. gondii* infection correlates with cyst load, Related to Figure 3

(A), (B) and (C) Schematic representation of the strategy used to generate transgenic strains used in this study and PCR or sequencing analysis to verify correct integration or locus disruption.

(D) Quantification of percentage of distance travelled in the open arm of an elevated plus maze of mice infected with *T. gondii* parasites deficient for protein export (ASP5-KO, MYR1-KO), with a low virulence strain (MyoJ-KO) or with a different apicomplexan parasite (*N. caninum*).

(E) Quantification of rearing and investigation in the open field.

Bars indicate mean ± SEM and each dot represents an individual. *p<0.05, **p<0.01, ***p<0.001, ****p<0.0001, black asterisks represent significant differences compared to uninfected mice and orange asterisks represent significant differences compared to ME49-infected mice.

Supplementary Table 4, related to Figure 3

Primers used in this study

P1	(5'-3')	cgctgagcagaaagcatagca
P2	(5'-3')	cgacgtcaggtttcatacaagc
P3	(5'-3')	gtcacttgttgtgccagttctac
P4	(5'-3')	cttgggggtcatcgcgacgaccagac
P5	(5'-3')	caaggactccgaatccgacgagtcactgaagcggccgctctagaactag
P6	(5'-3')	cacggtgagacgatgttccatcgttctgtggcggaagatccgatcttgc
P7	(5'-3')	gccggtaccgcgactttatccagttgcccg
P8	(5'-3')	gcgactagtgcggaagatccgatcttgc
gRNA1	(5'-3')	GAATGCGGAAGCAGGTTTCTTgttttagagctagaaatagc
gRNA2	(5'-3')	GCCGGGTCGGTGTATCCGACgttttagagctagaaatagc
gRNA3	(5'-3')	GACATCCACAGAACTTACTTgttttagagctagaaatagc
gRNA-4883	(5'-3')	aacttgacatccccatttac

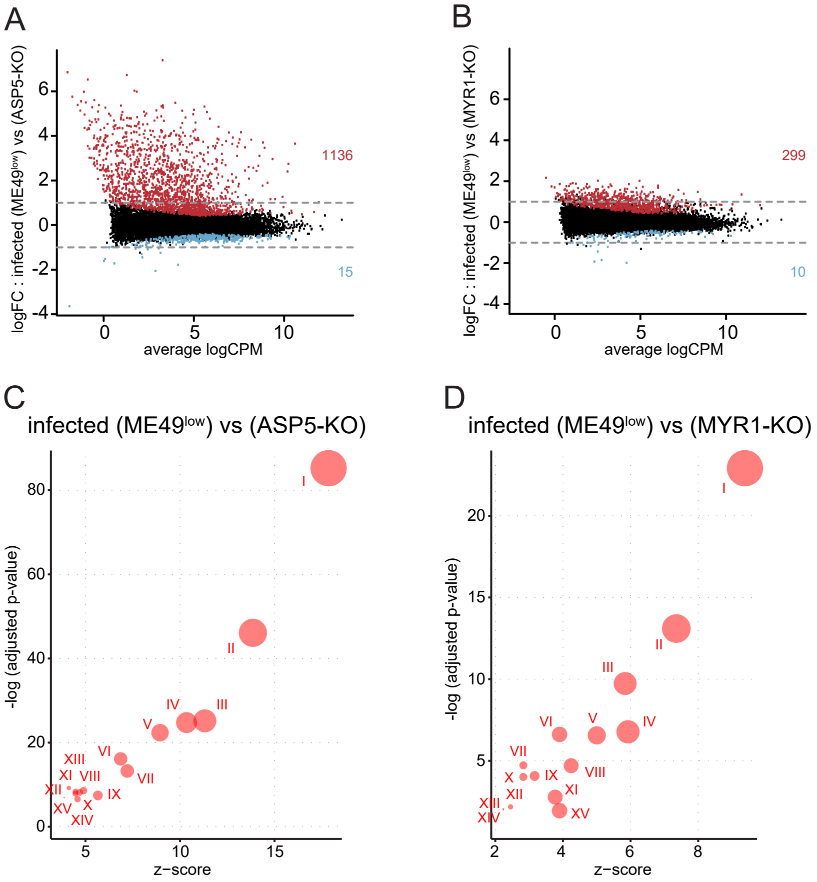

Upregulated pathways (C)

I Immune System
II Innate Immune System
III Adaptive Immune System
IV Neutrophil degranulation
V Cytokine Signaling in Immune system
VI Immunoregulatory interactions between a Lymphoid and a non−Lymphoid cell
VII Signaling by Interleukins
VIII Generation of second messenger molecules
IX Costimulation by the CD28 family
X Antigen processing−Cross presentation
XI DAP12 interactions
XII DAP12 signaling
XIII Toll−like Receptor Cascades
XIV GPVI−mediated activation cascade
XV TCR signaling

Upregulated pathways (D)

I Immune System
II Innate Immune System
III Neutrophil degranulation
IV Adaptive Immune System
V Immunoregulatory interactions between a Lymphoid and a non−Lymphoid cell
VI Cytokine Signaling in Immune system
VII Generation of second messenger molecules
VIII Signaling by Interleukins
IX TCR signaling
X Chemokine receptors bind chemokines
XI Class A/1 (Rhodopsin−like receptors)
XII Interleukin−2 family signaling
XIII Phosphorylation of CD3 and TCR zeta chains
XIV PD−1 signaling
XV GPCR ligand binding

E

Top 10 genes contributing to:

PC1		PC2		PC3	
+	-	+	-	+	-
Gnb2l1	Dclk1	Arhgap29	Pmm1	Mcts1	Upf1
Arrb2	Arhgef9	0610030E20Rik	Sec14l2	Ap3s1	Zfp865
Rgs19	Ttc3	Mfsd7b	Matk	Sumo1	Zswim8
Pctp	Lrrtm3	Cblb	Ttc9b	Ptges3	Ddx23
Casp6	Cdk14	Atl3	Ramp1	Nap1l1	Trpc4ap
Prdx6	Gabrb3	Tmem87b	Abhd17a	Ugp2	Sbf1
Eef1a1	Kidins220	Osbpl3	Stard10	Immp1l	Cnot3
AF251705	Nmnat2	Tgfbr3	Dgcr6	Cops5	Tnrc18
Rpl10	Inpp4a	Socs2	2210016L21Rik	Apoo	Map1s
Rplp0	Sh3gl2	Rnf111	Nudc	Rpap2	Flad1

Figure S4 – Host brain transcriptome shows sustained immune response in the CNS, Related to Figure 4
(A) and (B) Differential expression (DE) analysis showing expression of all detectable genes between ME49low-infected and ASP5-KO-infected (A) or MYR1-KO-infected (B). Red dots indicate upregulated genes in ME49-infected mice, blue dots indicate downregulated genes (FDR <0.05. Dashed lines indicate the threshold of a 2x fold change (FC).
(C) and (D) Pathway enrichment analysis for up-regulated genes in mice infected with *T. gondii* ME49low compared to ASP5-KO (C) or MYR1-KO (D).
(E) Top ten genes contributing positively and negatively to PC1, PC2 and PC3.

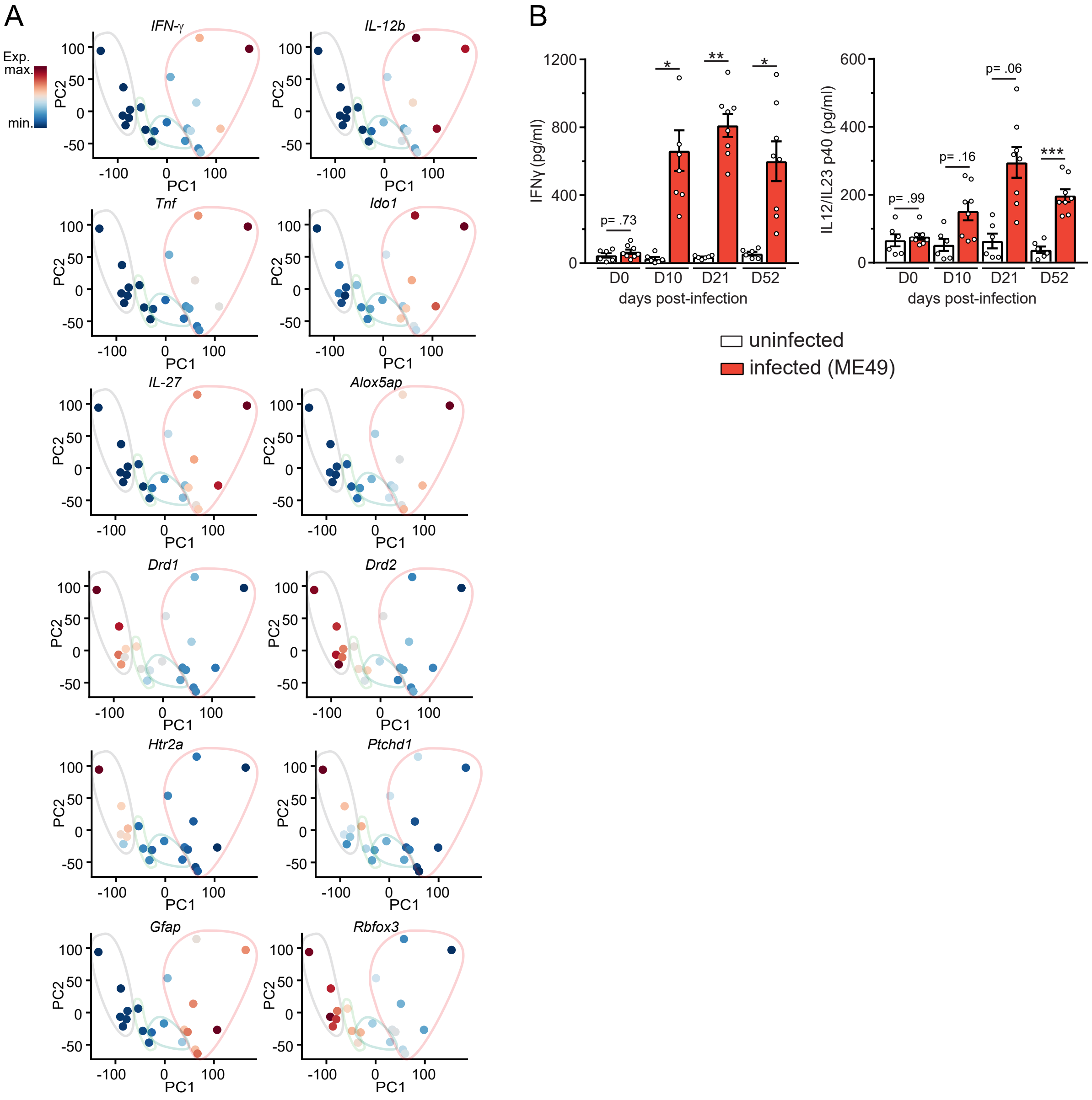

Figure S5 – Association of gene expression levels with infection and behavior, Related to Figure 5

(A) Relative gene expression levels representative of the up- and down-regulated pathways, displayed on a 2D PCA generated with all gene expression values (same as in Figure 4A). Ovals surround uninfected mice (grey) and mice infected with a parasite strain of the same genotype (colors).

(B) Concentration of IFN-γ and IL-12/IL-23 p40 in the plasma at 0, 10, 21 and 52 days postinfection in ME49-infected mice.

Bars indicate mean ± SEM and each dot represents an individual. *$p<0.05$, **$p<0.01$.

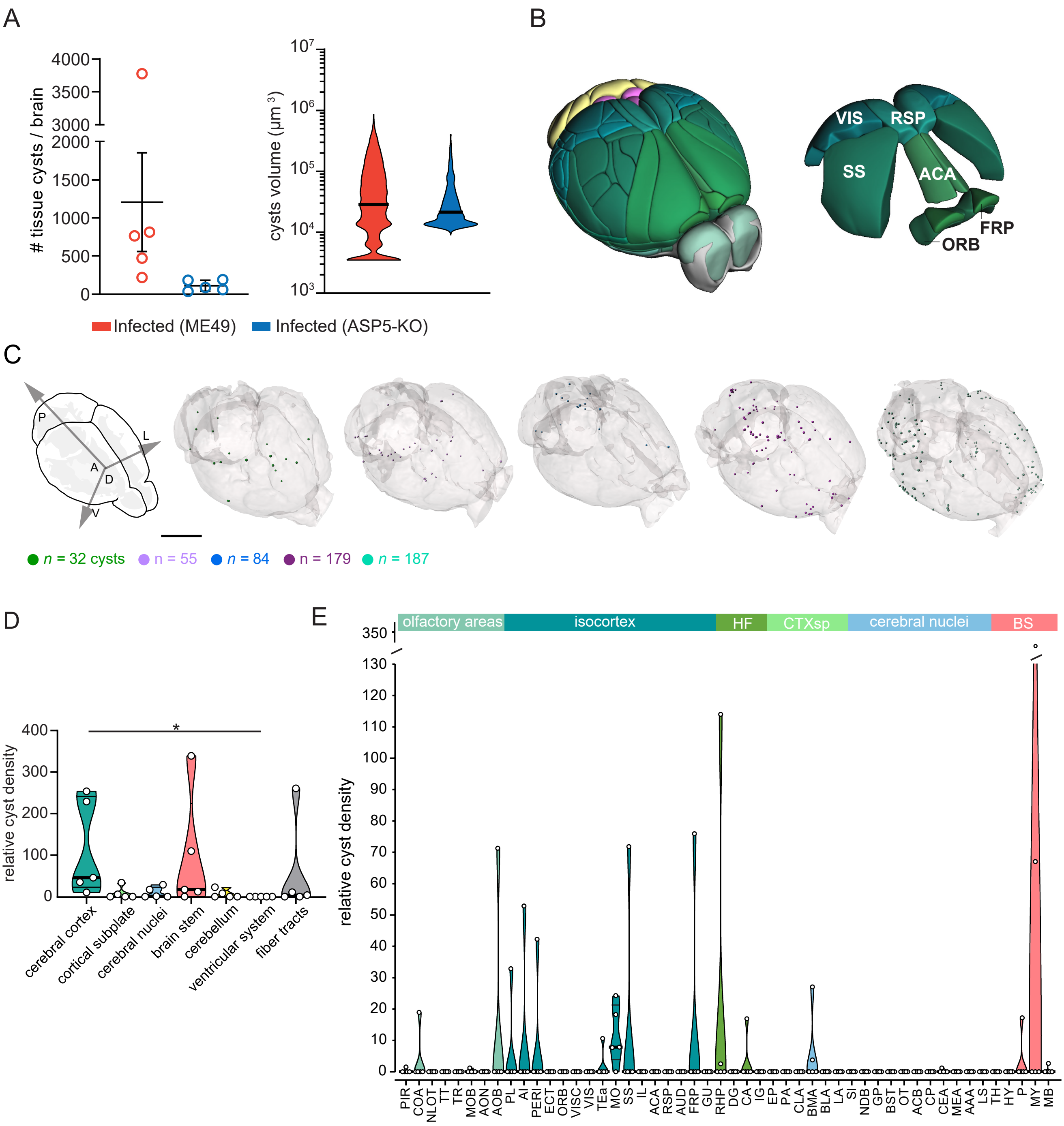

Figure S6 – Cartography of *T. gondii* cysts in the mouse brain, Related to Figure 6

(A) Cyst counts and volume obtained after segmentation, from all individuals infected with ME49-GFP or ASP5-KO-GFP.

(B) 3D illustration of subregions with highest relative density (percentage of cysts in a region / volume of the region (mm3)) of cysts in ME49-infected mice.

(C) 3D rendering of CLARITY-processed brains showing colorized *T. gondii* ASP5-KO-GFP cysts. The size of the cysts was purposely made uniform. Scale bar, 5 mm.

(D) Relative cyst density (percentage of cysts in a region / volume of the region (mm3)) in different regions of the brains of *T. gondii* ASP5-KO-GFP-infected mice.

(E) Distribution of the relative cyst density within subregions of the CNS in *T. gondii* ASP5-KO-GFP-infected mice. For abbreviations, see Table S4.

Bold bars indicate the median and thinner bars the quartiles. Each dot represents an individual.

Supplementary Table 5, related to Figure 6

Abbreviations of brain regions

**OLF**	**olfactory areas**
PIR	piriform area
COA	cortical amygdalar area
NLOT	nucleus of the lateral olfactory tract
TT	taenia tecta
TR	postpiriform transition area
MOB	main olfactory bulb
AON	anterior olfactory nucleus
AOB	accessory olfactory bulb
**ISO**	**isocortex**
PL	prelimbic area
AI	agranular insular area
PERI	perirhinal area
ECT	ectorhinal area
ORB	orbital area
VISC	visceral area
VIS	visual areas
TEa	temporal association areas
MO	motor areas
SS	somatosensory areas
IL	infralimbic areas
ACA	anterior cingulate area
RSP	retrosplenial area
AUD	auditory areas
FRP	frontal pole, cerebral cortex
GU	gustatory areas
**HF**	**hippocampal formation**
RHP	retrohippocampal region
DG	dentate gyrus
CA	ammon's horn
IG	induseum griseum
**CTXsp**	**cortical subplate**
EP	endopirifrom nucleus
PA	posterior amgdalar nucleus
CLA	claustrum
BMA	basomedial amygdalar nucleus
BLA	basolateral amygdalar nucleus
LA	lateral amygdalar nucleus
SI	substantia innominata
**CNU**	**cerebral nuclei**
NDB	diagonal band nucleus
GP	globus pallidus
BST	bed nuclei of the stria terminalis
OT	olfactory tuberle
ACB	nucleus accumbens
CP	caudoputamen
CEA	central amygdalar nucleus
MEA	medial amygdalar nucleus
AAA	anterior amygdalar area
LS	lateral septal nucleus
**BS**	**brain stem**
TH	thalamus
HY	hypothalamus
P	pons
MY	medulla
MB	midbrain
